# Hep3Gel: A Shape-Shifting Extracellular Matrix-Based, Three-Dimensional Liver Model Adaptable to Different Culture Systems

**DOI:** 10.1021/acsbiomaterials.2c01226

**Published:** 2022-12-16

**Authors:** Giuseppe Guagliano, Cristina Volpini, Lorenzo Sardelli, Nora Bloise, Francesco Briatico-Vangosa, Antonia Icaro Cornaglia, Silvia Dotti, Riccardo Villa, Livia Visai, Paola Petrini

**Affiliations:** † Department of Chemistry, Materials, and Chemical Engineering “G. Natta”, Politecnico di Milano, Piazza Leonardo da Vinci 32, 20133Milan, Italy; ‡ Molecular Medicine Department (DMM), Center for Health Technologies (CHT), UdR INSTM, University of Pavia, 27100Pavia, Italy; § Department of Public Health, Experimental and Forensic Medicine, Histology and Embryology Unit, University of Pavia, 27100Pavia, Italy; ∥ National Reference Center for Alternative Methods, Welfare and Care of Laboratory Animals, Istituto Zooprofilattico Sperimentale della Lomabardia ed Emilia Romagna, 25124Brescia, Italy; ⊥ Medicina Clinica-Specialistica, UOR5 Laboratorio Di Nanotecnologie, ICS Maugeri, IRCCS, Pavia, Via Boezio, 28-27100Pavia, Italy; # Interuniversity Center for the Promotion of the 3Rs Principles in Teaching and Research (Centro 3R), Università di Pavia Unit, 27100Pavia, Italy; ¶ Interuniversity Center for the Promotion of the 3Rs Principles in Teaching and Research (Centro 3R), Politecnico di Milano Unit, 20133Milan, Italy

**Keywords:** 3D cell cultures, 3D bioprinting, alginate, bioink, decellularized hepatic tissue, internal crosslinking

## Abstract

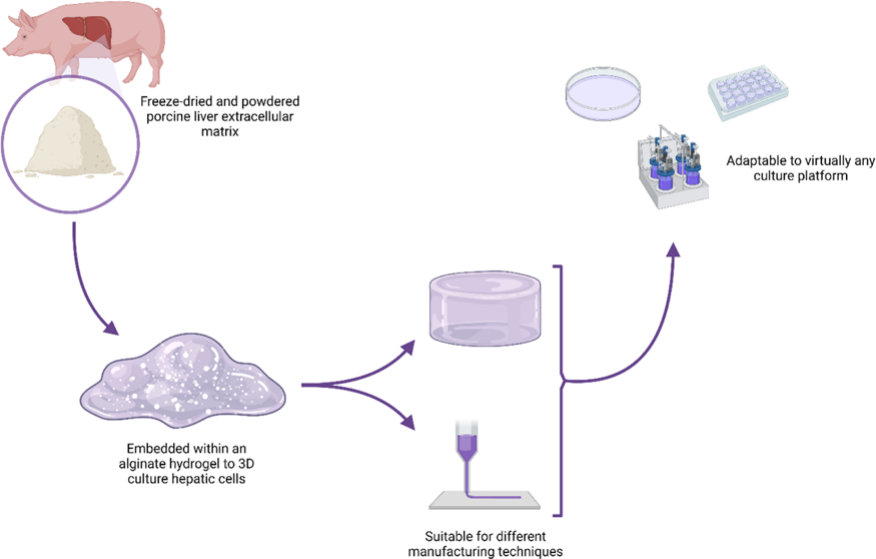

Drug-induced hepatotoxicity is a leading cause of clinical trial withdrawal. Therefore, in vitro modeling the hepatic behavior and functionalities is not only crucial to better understand physiological and pathological processes but also to support drug development with reliable high-throughput platforms. Different physiological and pathological models are currently under development and are commonly implemented both within platforms for standard 2D cultures and within tailor-made chambers. This paper introduces Hep3Gel: a hybrid alginate–extracellular matrix (ECM) hydrogel to produce 3D in vitro models of the liver, aiming to reproduce the hepatic chemomechanical niche, with the possibility of adapting its shape to different manufacturing techniques. The ECM, extracted and powdered from porcine livers by a specifically set-up procedure, preserved its crucial biological macromolecules and was embedded within alginate hydrogels prior to crosslinking. The viscoelastic behavior of Hep3Gel was tuned, reproducing the properties of a physiological organ, according to the available knowledge about hepatic biomechanics. By finely tuning the crosslinking kinetics of Hep3Gel, its dualistic nature can be exploited either by self-spreading or adapting its shape to different culture supports or retaining the imposed fiber shape during an extrusion-based 3D-bioprinting process, thus being a shape-shifter hydrogel. The self-spreading ability of Hep3Gel was characterized by combining empirical and numerical procedures, while its use as a bioink was experimentally characterized through rheological a priori printability evaluations and 3D printing tests. The effect of the addition of the ECM was evident after 4 days, doubling the survival rate of cells embedded within control hydrogels. This study represents a proof of concept of the applicability of Hep3Gel as a tool to develop 3D in vitro models of the liver.

## 1 Introduction

The liver is the largest organ of the human body and can be considered a major crossroad of human physiology. It carries out more than 500 viable functions, including glucose storage and delivery, lipid processing, blood filtration, and drug metabolism, and it also concurs with the maintenance of the systemic equilibrium through the cross-talk with other organs (e.g., gut–liver–brain axis, and liver–thyroid axis).^1−3^

In the past years, numerous in vitro models of the liver have been proposed, not only for fundamental research but also to support pharmaceutical development with reliable high-throughput platforms.^4,5^ In this context, two-dimensional models of the liver, intended as monolayers of hepatic cells grown on flat substrates such as Petri dishes or multiwell plates, were the first platforms exploited to evaluate the hepatotoxic response to the administration of active pharmaceutical ingredients in vitro. These types of models, which can rely both on primary cells and cell lines, thus providing robust and reproducible results, led the way for the production of more complex models.^6,7^ Up to now, some recent models have been based on three-dimensional cultures, where the cells are cultured within matrices mimicking the hepatic extracellular environment.^8^ These platforms allow not only to reproduce the in vivo-like spatial organization of cells but also the interactions occurring between cells and the extracellular environment.^9,10^ Moreover, culturing cells within a tridimensional construct provides them with a set of chemical and mechanical stimuli, similar to those experienced in vivo, which significantly enhances cell proliferation while mitigating the dedifferentiation phenomenon observable when culturing cell monolayers.^11^

Due to the soft nature of the liver itself, three-dimensional hepatic models are generally produced by embedding cells within hydrogels of both natural and synthetic origin since their mechanical properties can be tuned to be comparable with the ones of the native tissue.^12^ These cellularized constructs can be cultured both in static and dynamic conditions, and depending on the features to be modeled, they are usually implemented either within commercially available cell culture supports or within custom-made devices, such as culture chambers of bioreactors or microfluidic chips.^13−15^ As a consequence, the final shape of the 3D culture has to be optimized as a function of the culture system. According to the final application, various manufacturing techniques can thus be exploited to produce differently shaped constructs, including homogeneous briquette-like hydrogels, spheroids, caps, or macroporous structures characterized by more complex geometries.^16−18^

In light of this, the main requirement for the developed material is to be a shape-shifter, intended as being able to adapt to virtually any culture system and condition, by being used either as a hydrogel able to self-spread, adapting to different shapes, or as a bioink, able to retain the shape of the extruded fiber for the extrusion-based 3D-bioprinting (EBB) of customized constructs.

We describe Hep3Gel, a hybrid alginate–extracellular matrix (ECM) hydrogel, specifically designed to flexibly in vitro model the hepatic environment. ECM derived from native organs is an excellent material since it can provide embedded cells with the proper physiological biochemical compounds.^19,20^ However, the ECM has been demonstrated not to be suitable to be used as a standalone material due to the loss of mechanical properties after extraction and processing. On the other side, alginate is well known for the tunability of its crosslinking kinetics, as well as for the possibility to tailor its rheological behavior while being able to sustain the viability of embedded cells.^21−23^ For this reason, when designing Hep3Gel, alginate was introduced to tune the rheological properties of ECM-based hydrogels, making up for its scarce structural properties.

## 2 Experimental Section

### 2.1 ECM Powder Production

**Figure 1:**
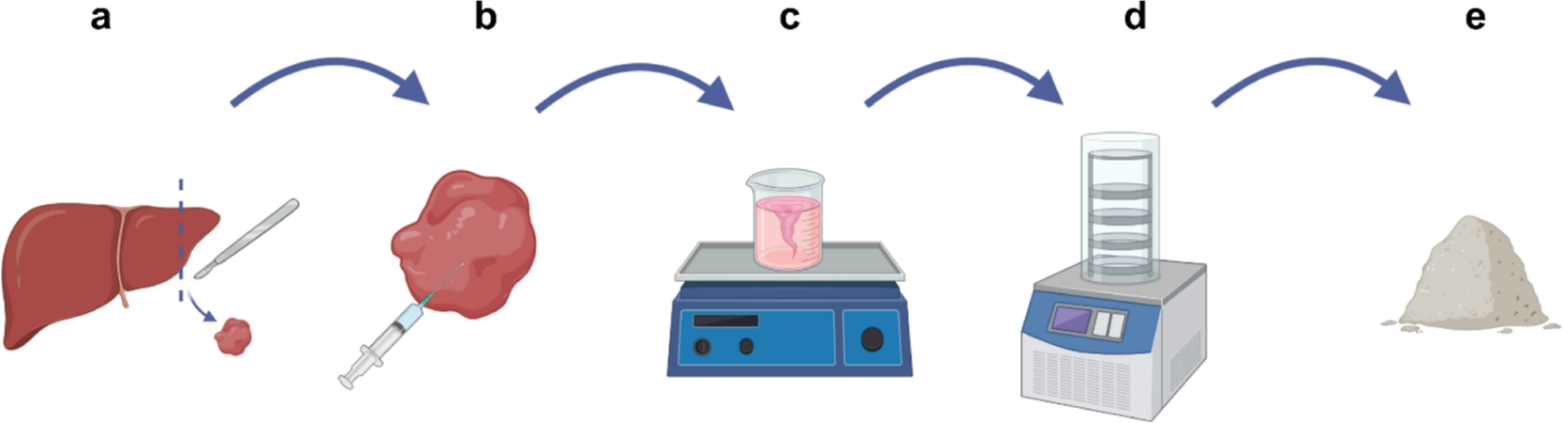
Processing steps to obtain pdECM powder and its intermediate products. (a) The porcine liver was cut into small pieces, (b) which were injected multiple times with the decellularization buffer, (c) orbitally shaken for 72 h, (d) freeze-dried, (e) and finally ground into a fine powder (created with BioRender.com).

Porcine liver decellularized ECM (pdECM) powder was obtained starting from porcine livers, provided by a local butcher. The organ was vigorously massaged to promote cellular lysis before starting the decellularization. The decellularization buffer was prepared by dissolving 1% (w/v) SDS (Bio-Rad, Cat. no. 1610302, Lot. no. 64064980, USA, CA) and (1% v/v) Triton X-100 (VWR Chemicals, EC no. 201-064-4, Lot. no. 17D034123, FR) in 1 M Tris-HCl buffer with pH 7.4 (tris(hydroxymethyl)-aminomethane: VWR Chemicals, EC no. 201-064-4, Lot. no. 17D034123, FR; HCl: Emprove, Lot. no. 231-595-7, EC), sterilely filtered, and stored at 4 °C. The liver was sectioned into small pieces of approximately 0.5–1 cm^3^ volume (Figure 1a). Each side of each liver piece was manually injected up to five times with 25 mL of decellularization buffer with approximately a 12.5 mL/min flow rate (Figure 1b). Liver cubes were then transferred into plastic bottles, submerged into the decellularization buffer, and left in a high-speed orbital shaker (≈140 rpm) for 72 h (Figure 1c). The decellularization buffer was refreshed every 24 h. pdECM was then recollected, washed with sterile deionized water, frozen at −80 °C overnight, and subsequently freeze-dried for 24 h (Figure 1d). Lyophilized pdECM was then ground into a fine powder using a commercial 1800 W cereal mill (CGOLDENWALL 322, CGOLDENWALL, CN) at 28,800 rpm after being cooled down at −120 °C (Figure 1e). More in detail, pdECM was submerged into liquid nitrogen in a pot, and immediately after complete evaporation, it was poured into the mill and processed for 3 min and then transferred again in the pot to be snap-frozen again at −120 °C. This cycle was repeated 10 times; then, pdECM was stored at −20 °C for further operations. The obtained powder was observed using an optical microscope (Leica DMi 1, Leica Camera AG, DE), and its granulometry was measured with FIJI.^24^

### 2.2 pdECM Characterization

#### 2.2.1 SDS and DNA Assay

SDS Detection & Estimation Reagent Kit (G-Biosciences, Cat. no. 786-129, USA) and DNeasy Blood & Tissue Kits (Qiagen, Cat. no. 69504, DE) were used according to the manufacturers’ instructions to detect and quantify the residual amounts of SDS and DNA, respectively.

#### 2.2.2 Sodium Dodecyl Sulfate–Polyacrylamide Gel Electrophoresis

pdECM powder was characterized from the point of view of preserved extracellular matrix components. Preliminary sodium dodecyl sulfate–polyacrylamide gel electrophoresis (SDS-PAGE) was performed by implementing a 12% (w/v) acrylamide gel (Bio-Rad, CA) to compare the protein content of samples of pdECM powder (PD) with one of the freeze-dried porcine liver native tissue samples (PN), by running a marker ranging from 17 to 245 kDa. Before running electrophoresis, both pdECM and native tissue samples needed to be solubilized. To this extent, two different lysis techniques have been compared. Half of the samples of each nature were placed in a microcentrifuge tube and immersed in urea buffer (25 mM) containing Tris-HCl pH 7.5 + 1% (w/v) SDS in 4.5 mM urea, vortexed for 1 min, and then sonicated (59 kHz, 24 °C, 15 min); this procedure was repeated until complete lysis. The remaining samples were subjected to the same lysis procedure, except that after sonication, they were also heated for 90 min at 60 °C. To evaluate the presence of type I collagen (Coll I) and fibronectin (Fn), an additional SDS-PAGE using 7% (w/v) acrylamide gel (Bio-Rad, CA) was performed both in nonreducing and reducing conditions (with/without mercaptoethanol). To this end, Coll I, used as the reference control for SDS-PAGE, was isolated with neutral salt from fetal bovine skin and purified by NaCl precipitation and DEAE chromatography.^25^ Fn used as the reference control for SDS-PAGE was purified from human plasma as previously reported.^26^ A Coomassie-stained protein gel (Bio-Rad, CA) was carried out for protein visualization. The residual presence of both Coll I and Fn was then investigated through western blot and dot-blot analyses.

#### 2.2.3 Western Blot

After sample fractionation on SDS-PAGE, the gels were electroblotted onto a nitrocellulose membrane (Hybond ECL, Amersham Pharmacia Biotech, Uppsala, SE). The membrane was treated with a solution containing 10% dried skimmed milk in 25 mM Tris-HCl, pH 7.4, washed, and then incubated with rabbit polyclonal anti-Coll I (diluted 1:1000) in TBS-T (TBS containing 0,5% Tween 20) provided by Dr. Larry W. Fisher (National Institutes of Health, Bethesda, MD, USA) or rabbit polyclonal anti-Fn antibody (diluted 1:1000 in TBS-T) and produced as previously described for 1 h at room temperature (RT).^27^ The membrane was washed three times for 10 min with TBS-T and incubated for 1 h with 10% milk containing secondary antirabbit IgG horseradish peroxidase conjugate. After several rounds of washing in TBS-T, the detection was performed with a western chemiluminescent HRP substrate (e.g., SuperSignal West Pico PLUS or SuperSignal West Atto Ultimate Sensitivity Substrate) (Thermo Fisher Scientific, Waltham, MA, USA) and revealed using an ImageQuant LAS4000 Imaging System (GE Healthcare Life Sciences, Pittsburgh, PA, USA). Band densitometry analysis was carried out with FIJI.

#### 2.2.4 Dot Blot

The amount of Coll I and Fn in the ECM of both PN and PD was detected by using a dot blot. In brief, an increasing amount of ECM–proteins (0.5, 0.7, 1.0, and 1.5 μg) were spotted on the nitrocellulose membrane (Amersham Hybond ECL, GE Healthcare Life Sciences) and air-dried. The nonspecific sites were blocked by soaking the membrane in 5% BSA (Sigma-Aldrich, US) in TBS-T for 1 h at RT. The membrane was then incubated overnight at 4 °C with a primary rabbit polyclonal antibody against Coll I (diluted 1:1000 in TBST) or anti-Fn. After extensive washing and incubation with a secondary HRP-conjugated antirabbit antibody, the membranes were developed following the same procedure applied in western blot analysis and ImageQuant LAS4000 Imaging System (GE Healthcare Life Sciences). The results were normalized to the calibration curve performed using increasing concentrations of Coll I and Fn purified as described previously.^26,28,29^

#### 2.2.5 ECM Component Quantification

To this end, data obtained from the dot blot were processed to quantify the residual amount of both Coll I and Fn in PN and PD. The Fastin Elastin Assay (F2000, Biocolor Ltd., #BB498-kit, UK) and Blyscan Sulfated Glycosaminoglycan (GAG) Assay (S1000, Biocolor Ltd., #BB492-kit, UK) were used according to producers’ protocols to quantify residual elastin and GAGs, respectively, in both freeze-dried PN and PD powder samples.

### 2.3 Hep3Gel Production

Hydrogels were produced by sequentially mixing different solutions and suspensions with coupled syringes, exploiting the alginate internal crosslinking mechanism. All of the following solutions and suspensions were prepared in a complete HepG2 culture medium, which is composed of Eagle’s minimum essential medium (EMEM) with Earl’s salts (EuroClone, Cat. no. ECB2071L, IT), 10% (v/v) fetal bovine serum (FBS) (EuroClone, Lot. no. EU-S021179, IT), 1% (v/v) Na pyruvate (EuroClone, Lot. no. EU-M00QU, IT), 1% (v/v) glutamine (EuroClone, Lot. no. EU-M0150017, IT), and 1% (v/v) penicillin–streptomycin (Lonza, Lot. no. 2MB027, BE). Alginate powder (Sigma-Aldrich, Lot. no. MKJC8027, US) was disinfected by multiple immersions in 96% (v/v) ethanol (Emprove, Lot. no. 1.00967.2500, EC) and air-dried overnight under a laminar flow hood. Autoclave sterilization was not performed since thermally treating sodium alginate can impair its macromolecular chain.^30^ Additionally, 96% (v/v) ethanol was preferred over the typical 70% (v/v) to prevent undesired partial dissolution due to the presence of a higher fraction of water, which also contributes to increasing the drying time of pdECM after disinfection.

To prepare Hep3Gel precursor solutions, disinfected alginate was slowly poured into a medium while magnetically stirring at RT, until reaching a final alginate concentration of 3.5% (w/v). pdECM powder was exposed to UV light under a laminar flow hood for 2 h to be disinfected. Once alginate was completely dissolved, and no inhomogeneities or aggregates were visible within the alginate solution (approximatively 3 h), disinfected pdECM was gently poured into it, up to a 1.4% (w/v) final concentration. The Hep3Gel precursor solution was thus magnetically stirred until reaching the macroscopic homogeneous dispersion of pdECM powder (approximately 6 h). In addition to Hep3Gel, two other types of hydrogels, one made with alginate and gelatin (GEL) and the other with only alginate (ALG), were produced as controls to study the effects induced by the presence of the ECM. To prepare the GEL precursor solution, gelatin (Sigma-Aldrich, Lot. no. G9382, US) was disinfected in the same way as alginate, before being dissolved (1.4% w/v) at 50 °C while stirring. Alginate (3.5% w/v) was added and dissolved after the gelatin solution cooled down to RT. On the other hand, the ALG precursor solution was prepared as the one of Hep3Gel but by avoiding the addition of pdECM powder. A 0.7% (w/v) suspension of CaCO_3_ was prepared after having disinfected CaCO_3_ in a 120 °C drying oven for at least 6 h. Four parts of the alginate-based solution and one part of the CaCO_3_ suspension were poured into different syringes. After having removed all air bubbles from the syringes, they were connected with a female–female Luer-lock connector and mixed 45 times at RT, reaching in this way a 0.02 M concentration of Ca^2+^ ions within the final volume of the hydrogel. One part of the complete medium was then poured into a syringe, and after having removed air bubbles, it was connected to the syringe containing the alginate-based solution and CaCO_3_ suspension and mixed 45 times at RT. The added amount of fresh medium is the volume that should host the cellular suspension when embedding cells within hydrogels; when producing noncellularized hydrogels, a fresh medium was added to reduce physical differences between noncellularized and cell-embedding hydrogels. Finally, the syringes Hep3Gel, GEL, and ALG precursor solutions + CaCO_3_ suspension + fresh medium were mixed 45 times at RT with a syringe containing one part of a 7% (w/v) sterilely filtered GDL (Sigma-Aldrich, Lot. no. SLCF8971, US) solution, aiming to lower the pH, and inducing the dissolution of CaCO_3_ the subsequent release of Ca^2+^ ions and, consequently, the beginning of the crosslinking reaction. Immediately after the addition of GDL, materials were extruded into 6-, 12-, and 24-well plates or loaded within 3 mL 3D-bioprinting cartridges (CELLINK, SE), depending on the experimental needs. The process to produce Hep3Gel and control materials is illustrated in detail in Figure S1. The maintenance of sterility during the productive process was evaluated after each step by plating each reagent and compound on Muller–Hinton agar plates (Sigma-Aldrich, Cat. no. 102097316, US), Luria–Bertani agar plates (Formedium, Cat. no. LMG0102, UK), and tryptic soy agar plates (Formedium, Cat. no. TSB0110, UK). To assess the absence of bacterial and fungal contamination, plates were then incubated at 37 °C for 3 days.

### 2.4 Immunohistochemical Analyses

Non-cell-loaded Hep3Gel samples were fixed in 10% (v/v) neutral buffered formalin for 24 h at RT. After washing them with tap water for 1 h, samples were dehydrated in graded alcohol as follows: immersion in 50% (v/v) ethanol for 1 h, in 70% (v/v) ethanol for 1 h, and in 80% (v/v) ethanol overnight. After this, the passaged samples were soaked in 95% (v/v) ethanol three times for 1 h each, immersed in 100% (v/v) ethanol three times for 1 h each, and finally washed with xylol for 15–45 min. Samples were then kept in hot paraffin wax at 56 °C overnight to be subsequently embedded within paraffin blocks. Slices of 7 μm height were produced with a microtome. Sections were placed into a 40 °C water bath until they were transferred to Superfrost Plus slides by flotation. Samples were deparaffinized with xylene and rehydrated through graded alcohols to deionized water and then washed with sterile phosphate-buffered saline for 10 min, thus being ready to be stained.

#### 2.4.1 Type I Collagen, Fibronectin, and Elastin

Samples were permeabilized with 0.1% Triton X-100 in PBS 1× for 1 h at RT, and then to block nonspecific staining between the primary antibodies and the tissue, sections were incubated with 1% horse serum in PBS for 30 min at RT. Primary antibodies were added against type I collagen (1:100, Merck Millipore), fibronectin (20 μg/mL), produced as previously described,^27^ and elastin (1:500; Sigma-Aldrich) diluted in 1% horse serum in PBS + 0.02% (v/v) Tween 20. Samples were incubated overnight at 4 °C with primary antibodies; then, hydrogels were incubated with polyclonal goat antirabbit Ig/HRP secondary antibody (1:150 Dako) (for type I collagen and fibronectin immunostaining) and with polyclonal rabbit antimouse Ig/HRP secondary antibody (1:150 Dako) (for elastin staining), both diluted in incubation buffer for 1 h at RT and protected from light. At the end of the incubation, 0.03% of DAB dissolved in Tris buffer + 0.02% H_2_O_2_ to reveal the precipitate. Lastly, tissues were dehydrated and cleared in xylene and mounted.

#### 2.4.2 Glycosaminoglycans

Slices were stained in 1% (w/v) Alcian Blue solution (Alcian Blue 8GX, Sigma-Aldrich, in 3% acetic acid, pH: 2.5) for 30 min and washed in running tap water for 10 min. Sections were rinsed in deionized water and counterstained with a 0.1% w/v Nuclear Fast Red solution for 5 min, washed in running tap water for 1 min, and dehydrated. Lastly, tissues were cleared in xylene and mounted.

#### 2.4.3 Imaging

Images from samples produced as in 2.4.1 and 2.4.2 were acquired using a confocal laser scanning microscope (Leica SP8, Leica Camera AG, DE) at 4× magnification. Images were acquired from different contiguous fields and then combined in a final image (thus illustrating an overall field of 5 mm × 6 mm) with the Leica proprietary software (LAS X, Leica Camera AG, DE).

### 2.5 Physical Characterization of Hep3Gel

After being produced, hydrogels were weighed, before and after being dried, characterizing them in terms of solid and liquid fractions. Hep3Gel and control hydrogels were submerged in a complete fresh medium and tested for stability in static culture conditions (37 °C, 95% humidity, 5% CO_2_) for up to 12 days.

Both Hep3Gel and control hydrogels were manually sectioned, and approximately 1 mm-thick slices were obtained from the top, cross section, and bottom planes of hydrogels. Slices were observed with a backlight optical microscope (BX60, Olympus, JP).

The viscoelastic properties of hydrogels were characterized using a modular rheometer (MCR 502e, Anton-Paar, AT). If not differently stated, all tests were performed adopting a double-plate geometry with 25 mm diameter plates (Anton-Paar, serial number: 52890). If not differently specified, the temperature was set to 37 °C and controlled with a Peltier plate and hood system. After ensuring that no slip occurred, the distance between plates was always set equal to 0.5 mm. The crosslinking kinetics of Hep3Gel, GEL, and ALG were characterized through time-sweep tests (0.5% shear strain) at a constant frequency of 1 Hz, up to 24 h from the beginning of the crosslinking. Since carrying out the whole time sweep at 37 °C could produce artifacts in the measurements due to the drying of the samples, the full-length tests were carried out at 25 °C. To study the influence of temperature on the crosslinking kinetics, sequential tests at 37 °C were carried out for 0–20 min, 40–60 min, 2:00–2:20 h, 4:00–4:20 h, and 23:40–24:00 from the beginning of the crosslinking on samples from coherent batches that were stored at 37 °C until the measurement. Further information related to the behavior of materials while crosslinking was acquired through the analysis of their viscosity profiles. To this end, the viscosities η of Hep3Gel, GEL, and ALG were measured from the beginning of crosslinking up to their gel point, with a rheometer mounting with a 50 mm double-plate geometry (Anton-Paar, serial number: 52530), implementing a constant shear rate , equal to 100 s^–1^.

Storage (*G*′) and loss (*G*″) moduli within the linear viscoelastic region were measured for each type of hydrogel at 37 °C, with a frequency-sweep test (0.5% shear strain) in the frequency range of 0.1–22.5 Hz. Homologous frequency-sweep tests were also carried out on both cellularized and noncellularized hydrogels. To this end, fully crosslinked hydrogels were immersed in a culture medium and tested at different time points (1, 4, 8, and 12 days), aiming to provide further information on their structural changes cultured.

### 2.6 Characterization of the Shape-Shifting Behavior

#### 2.6.1 Hep3Gel for Self-Spreading 3D Matrices

The ability of engineered materials to adapt to the shape of the desired mold, aiming to produce a homogeneous tridimensional matrix, was characterized by evaluating the ability of the materials to self-spread on a surface. From a qualitative point of view, materials were extruded from a syringe into 24-, 12-, and 6-well plates at the beginning of the crosslinking. After the gelation was completed, hydrogels were gently extracted from the multiwell plates, macroscopically observed, measured in height and diameter, and cross-sectioned to evaluate the presence of bubbles or inhomogeneities in their core regions. Hep3Gel matrices were then observed with a transmitted light microscope (Leica DMi 1, Leica Camera AG, DE), aiming to characterize the dispersion of the pdECM powder throughout the hydrogel.

Quantitatively, the spread-ability of Hep3Gel and control materials was characterized by deposing 100 μL drops of each material on a polystyrene Petri dish every 30 min from the beginning of the crosslinking to the gel point. High-quality (4K, 60 FPS) videos of the drop spreading were filmed using a stabilized iPhone 12 Pro Max (Apple Inc., CA, USA). The diameters of the spread drops were measured with FIJI at the equilibrium, allowing us to calculate the spreading ratios (*S*_0_) (eq 1).
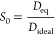
1

where *D*_eq_ is the drop diameter at the equilibrium and *D*_ideal_ is the diameter of a spherical drop of equal volume. Then, considering that a drop spreading on a flat surface takes the shape of a spherical shell, it was possible to estimate the minimum height of the layer obtainable with each material at each of the analyzed time points.

Contextually, the interdependence between the advancing of the crosslinking and the ability of materials to self-spread was studied by measuring the viscosity of crosslinking solutions through shear rate amplitude sweep. These tests were carried out implementing a 50 mm double-plate geometry (Anton-Paar, serial number: 52530) with the shear rate  ranging between 1 and 100 s^–1^.

### 2.7 Hep3Gel for 3D-Bioprinted Matrices

First, the possibility to use Hep3Gel as a bioink for EBB applications was studied a priori by the means of two specific rheological tests. These analyses were performed on a fully crosslinked hydrogel, 24 h after the beginning of the gelation. In the first test, the presence of yield stress was assessed with shear stress amplitude sweep in oscillation, with the amplitude ranging from 0.1 to 100 Pa at a constant frequency of 1 Hz. Then, a recovery test in the oscillatory regime was carried out by measuring *G*′ and *G*″ for all the time points considered at three strain amplitudes (0.5%, 100%, and again 0.5%), applied in sequence for 100, 200, and 100 s, respectively, at a constant frequency of 1 Hz.

A pneumatic bioprinter (INKREDIBLE+, CELLINK, SE) was then used to experimentally evaluate the printability of fully crosslinked hydrogels with standard 22G cylindrical steel needles with a 1.25 cm length (CELLINK, SE). The minimum extrusion pressure, intended as the minimum pressure to extrude a continuous filament, was determined for each material. This pressure was then implemented to print fibers with a length of 3 cm and 20 × 20 × 1 mm^3^ bilayered squares, characterized by a grid pattern infill, with a 25% density. Both fibers and grids were fabricated by implementing a 7 mm/s printhead speed. The relaxation of the fibers following the extrusion was evaluated by calculating the spreading factor (*S*), according to eq 2.
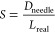
2where *D*_needle_ is the internal diameter of the needle and *L*_real_ is the real average diameter of the fiber, measured in 10 random points of each of 5 different fibers for each material. The quality of the extruded fibers was quantified by calculating the uniformity coefficient (*U*) (eq 3).
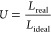
3where *L*_ideal_ is the diameter of a straight and homogeneous ideal fiber, estimated by multiplying *D*_needle_ for the spreading factor.

The quality of the grids was evaluated by calculating the perimeter coefficient (*P*_e_) and the pore coefficient (*P*_r_), according to eqs 4 and 5, respectively.
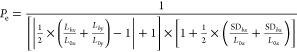
4
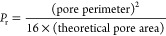
5where *L*_0*x*_ and *L*_0*y*_ are the theoretical lengths of sides, as provided to the printing system, *L*_*bx*_ and *L*_*by*_ are the average lengths of the printed sides, and SD_*bx*_ and SD_*by*_ are their standard deviations.

These data were subsequently combined as in eq 6 to compute the printability coefficient (*P*), to generally quantify the printability of hydrogels, as previously described.^31^
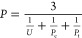
6

### 2.8 Cell Culturing and Embedding

Two vials containing approximately 2 million HepG2 cells at passage 99 were kindly provided by “Istituto Zooprofilattico Sperimentale della Lombardia e dell’ Emilia-Romagna”. Cells were thawed, counted in a hemocytometer by means of the trypan blue (Sigma-Aldrich, Lot. no. RNBD9396, US) exclusion assay, and seeded in a 6-well plate with a seeding ratio of approximately 300,000 cells/well. The medium used to culture cells was composed of EMEM with Earl’s salts (EuroClone, Cat. no. ECB2071L, IT), 10% (v/v) FBS (EuroClone, Lot. no. EU-S021179, IT), 1% (v/v) Na pyruvate (EuroClone, Lot. no. EU-M00QU, IT), 1% (v/v) glutamine (EuroClone, Lot. no. EU-M0150017, IT) and 1% (v/v) penicillin–streptomycin (Lonza, Lot. no. 2MB027, BE), and was refreshed every 48 h. Cells were expanded up to passage 6 and then cryopreserved (10^6^ cells/cryovial) in complete medium + 10% (v/v) DMSO (Sigma-Aldrich, Lot. no. SZBD2870V, US). To be embedded within hydrogels, cells were expanded, detached with trypsin (Lonza, Lot. no. 4MB146, BE), counted, suspended in a fresh medium, and embedded within gels as described in 2.3 at a final density of 3 × 10^6^ cells/gel. Hydrogels were then submerged with 2 mL of fresh medium and incubated at 37 °C, 95% humidity, and 5% CO_2._

### 2.9 Spatial Distribution of Embedded Cells

Cells were stained according to the producer’s instructions with Hoechst-33342 (Sigma-Aldrich, Cat. no. 875756-97-1, US), then resuspended in a fresh medium, counted, and embedded within Hep3Gel, GEL, and ALG precursor solutions at a final concentration of 5 × 10^6^ cells/mL. After the addition of GDL, samples of each material were extruded into microscopy-specific culture chambers (Ibidi, μ-Slide 8 well, Cat. no. 80806, DE). Samples were observed from their top, their bottom, and their cross-sectional plane by CLSM (Leica SP8, Leica Camera AG, DE) immediately after the beginning of the crosslinking and immediately after the gel point. The distribution of cells embedded within the matrices was quantitatively evaluated with FIJI before and after the gel point by analyzing the percentage of areas occupied by cells on different planes from the top, middle, and bottom layers of each material, respectively. To this end, CLSM images were converted to an 8-bit format, and their threshold was adjusted to display cells in white and the matrix in black while reducing the background noise. At this point, it was possible to measure the black fraction of the image and obtain the fraction occupied by cells by subtracting this value from the total. A qualitative overview of the distribution of cells within hydrogels was obtained through the 3D reconstruction using the Leica proprietary software (LasX, Leica Camera AG, DE).

### 2.10 Cell Viability Analyses

Cell viability after encapsulation was evaluated both quantitatively and qualitatively after 4 h, 1, 2, 3, 5, and 8 days from the encapsulation. Viability data are reported as survival rates normalized as a percentage of the number of viable cells 4 h after the encapsulation.

#### 2.10.1 Quantitative Cell Viability Analysis

For the quantitative evaluation, hydrogels were dissolved with a 50 M sterile solution of Na-citrate (Sigma-Aldrich, Lot. no. BCBW9965, US); then, each dissolved gel was poured in a 15 mL centrifuge tube and centrifuged (1200 rpm, 3 min). Cells were thus pelleted, retrieved, and suspended in 500 μL of a FBS-free medium. 100 μL of cellular suspensions were transferred to a 96-well plate, and the metabolic activity was quantified by the MTT assay (Sigma-Aldrich, Lot. no. MKBS4732V, US) according to the manufacturer’s instructions. The results were obtained through OD_570-630_ spectrophotometry analysis.

#### 2.10.2 Qualitative Cell Viability Analysis

Qualitative viability analyses relied on CLSM (Leica SP8, Leica Camera AG, DE) observations. Each gel was longitudinally sectioned and stained with Syto9 and propidium iodide (Invitrogen, Cat. no. L7012, MA, USA) according to protocols provided by the producer. Residual dyes were washed away with PBS 1× by pipetting. Gels were observed in their side and core regions, with both 20× and 40× immersion objectives. Coherently with the nature of used dyes, images were acquired implementing 488–500 and 535–570 nm as excitation–emission wavelengths for Syto9 and propidium iodide, respectively. A Leica proprietary LasX 3D visualization tool was used to three-dimensionally reconstruct CLSM images.

### 2.11 Statistical Analyses

All the experiments were performed technically in triplicate in at least two independent batches. Data are expressed as mean ± standard deviation. Standard deviations are displayed as a bar or as a range depending on the graph type, are not represented, and errors are graphically smaller than the symbols. Normality tests were performed to investigate the Gaussian distribution of data, and *t*-tests or Mann–Whitney tests were used to compare two groups of data, depending on whether them being normally distributed or not. Data representation and statistical analyses were performed using GraphPad Prism (GraphPad Software, Inc., CA, US), release 9.0.2, and different degrees of significance were considered (ns *p* > 0.05, *p < 0.05, **p < 0.01, ***p < 0.001).

## 3 Results and Discussion

### 3.1 Characterization of the pdECM Powder

**Figure 2:**
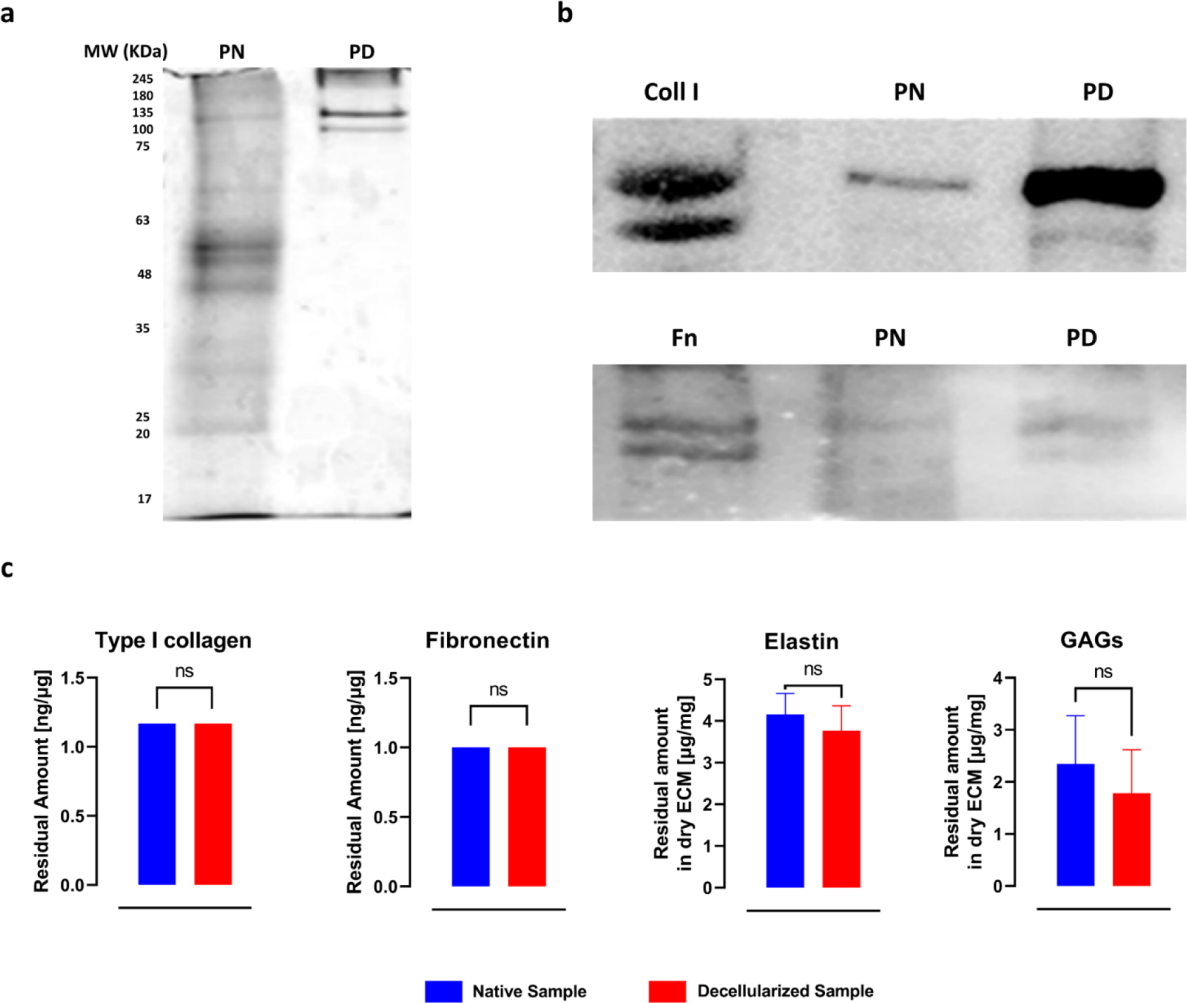
Characterization and quantification of the decellularized ECM components. (a) Representative images of nonreducing condition SDS-PAGE performed as indicated in 2.2.2; equal total protein was loaded for each sample (20 μg). (b) Representative western blot analysis of Coll I and Fn content probed with rabbit anti-coll I, and anti-Fn antibody, respectively. PN and PD stand for porcine native and porcine decellularized samples, respectively. (c) Quantification of the residual components and comparison with the amount of the native sample. (d) 10× magnification of immunohistochemical analyses of Hep3Gel slices, staining different components of the ECM; the scale bars correspond to 100 μm.

The ECM obtained from native organs is, in principle, the golden standard to reproduce the composition of the biochemical niche for the production of both in vitro devices and regenerative medicine organoids.^32,33^ Various approaches to the implementation of the ECM within 3D cell cultures have been developed in the past few years. Concisely, once an organ has been decellularized, there are two different classes of approaches to process the tissue to produce a soluble ECM. The most common strategies rely on enzymatically digesting ECMs.^34^ Enzymes such as pepsin and papain can quickly transform the ECM from an insoluble solid to a water-soluble fluid, which can be easily and homogeneously mixed with hydrogel precursor solutions.^35^ Unfortunately, enzymatic digestion is an extremely aggressive procedure and heavily contributes to the further degradation of ECM components obtained from the decellularization process, thus limiting their beneficial impact on the cultured cells.^36^ Less commonly, the ECM can be freeze-dried, powdered, and included as a microparticulate within a hydrogel, prior to crosslinking.^35^ This approach does not rely on reactions that can induce a systematic and aggressive denaturation of the ECM components, thus preserving the functionality of physiological interaction sites. The described decellularization procedure relies on the combination of different strategies available in the literature.^37^ It was tuned aiming to minimize the processing time. The results indicate that the process has a high degree of reproducibility among different batches (*p* < 0.05 for all the considered parameters) and allows obtaining easy-to-store and ready-to-use pdECM powder with a production process of overall 5 days.^38^ pdECM powder is characterized by an average granulometry of 147.9 ± 73.9 μm as shown in the Supporting Information (Figure S2) and has a residual presence of 0.3 μg of SDS per 1 mg of pdECM powder and DNA residual (0.6 ng/μg) was reduced from the amount present in the native liver (18 ng/μg). These concentrations do not represent a risk for cellular survival and proliferation within Hep3Gel and are comparable with results obtained through well-established whole-organ decellularization approaches.^39^ Once the presence of potentially toxic compounds was excluded, pdECM powder was tested to evaluate the aftermaths of decellularization on potentially beneficial biomolecules. The reported results refer to the lysis process without thermal treatment. No difference was reported between the two lysis techniques. After the decellularization procedure, higher-molecular-weight proteins are preserved, showing that the large-molecular-weight ECM–proteins have been enriched in the acellular matrix (Figure 2a). Detailed pictures of the preliminary SDS-PAGEs are reported in the Supporting Information (Figures S3 and S4). Heavier extracellular peptides such as type I collagen and fibronectin were preserved too (Figure 2b). Quantitative results (Figure 2c) showed that the whole procedure to produce pdECM powder does not significantly impact the original amount of ECM compounds.

The preservation of all of these ECM compounds after decellularization is crucial for the fate of embedded cells.^40,41^ Collagenous proteins and elastin are fibrillar components that mainly exert mechanical functions. They can interact with each other, providing the physiological microenvironment with adequate tensile strengths, resilience, and elastic recoil.^40,42^ Conversely, fibronectin plays a pivotal functional role. It can interact both with cellular integrins and with other ECM components such as collagen and GAGs. Additionally, fibronectin is directly linked with the regulation of major biochemical pathways, including cell adhesion, proliferation, migration, and differentiation.^43,44^ Last but not least, GAGs are highly hydrophilic molecules responsible for cell hydration and ECM assembly.^45^ Considering the impact that these macromolecules can have on the fate of cultured cells, together with the similarities between the macromolecular composition of human and porcine livers, the porcine hepatic ECM was chosen as a main component of Hep3Gel.^46−48^

### 3.2 Characterization of Hydrogels

**Figure 3:**
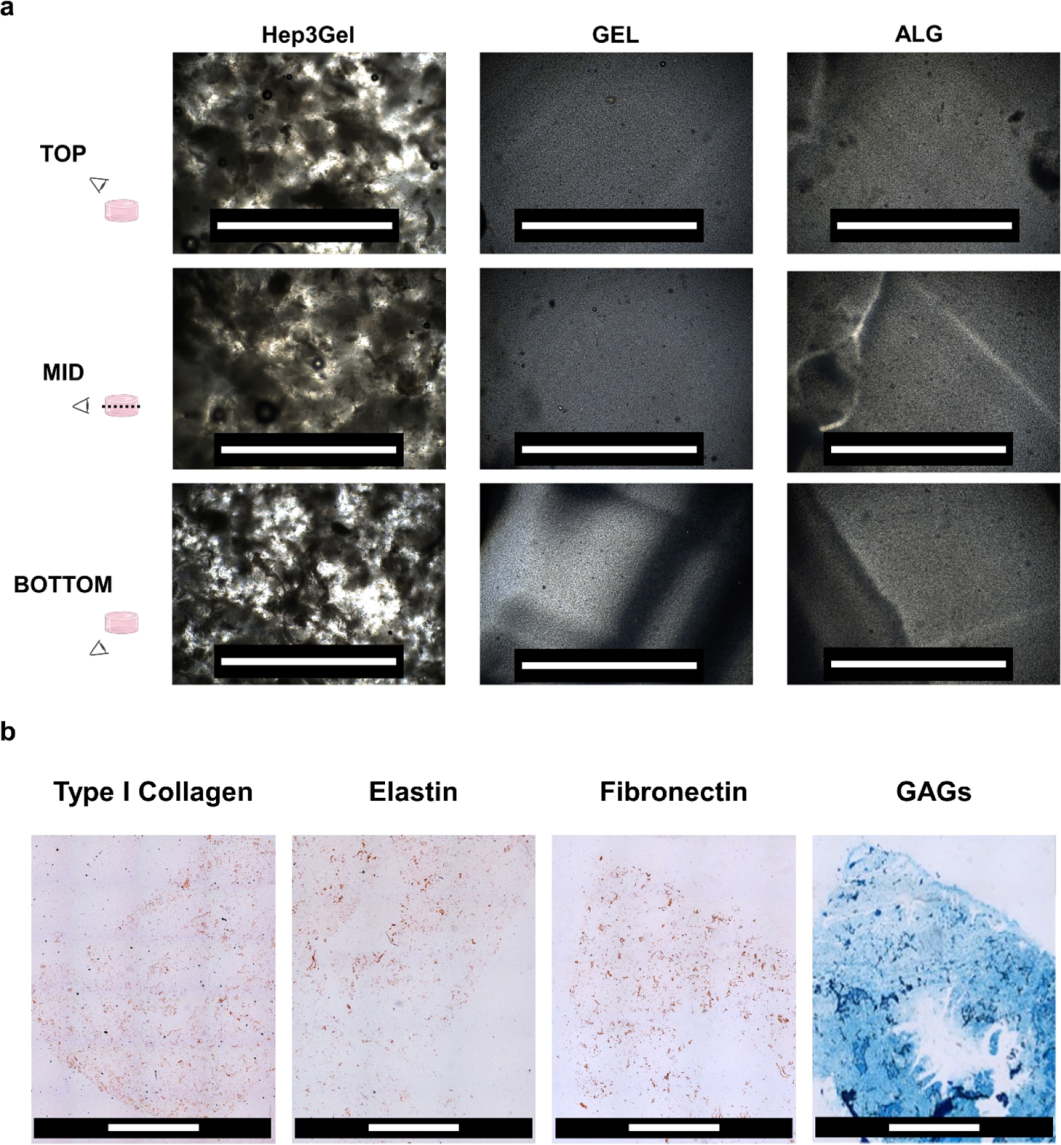
Distribution of the ECM within hydrogels. (a) Backlight microscopies acquired with a 4× magnification in different planes of each sample. In Hep3Gel, it is possible to see that the pdECM powder is homogeneously distributed throughout the volume of the material. Shadows and inhomogeneities that are observed in samples of GEL and ALG are due to the rhymes of resection, which was manually performed with a scalpel. The scale bar is equal to 500 μm. (b) Immunohistochemical images of Hep3Gel after having stained type 1 collagen, elastin, fibronectin, and glycosaminoglycans. Images were obtained after the “mosaic-like” reconstruction performed by the Leica software on different 4× magnification images from contiguous fields. The final images refer to a 5 × 6 mm^2^ field. The scale bar is equal to 2 mm.

Macroscopically, hydrogels are smooth and homogeneous and do not display bubbles or slits. Backlight macroscopic observation of Hep3Gel revealed the presence of two distinct phases, a dispersing one (alginate hydrogel matrix) and a dispersed one (pdECM), that is homogeneously distributed among the top, cross-sectional, and bottom planes. These two phases were not appreciated in GEL and ALG, as shown in Figure 3a. Coherently with the homogeneous distribution of pdECM powder, mosaic-like digitally reconstructed images from immunohistochemical analyses highlighted the homogeneous spatial distribution of ECM components (Figure 3b).

Examples of the single images acquired at a 4× magnification are reported for each of the studied macromolecules in Figure S5. Apart from the importance of immunohistochemical images in underlining the preservation of ECM compounds, also revealing their distribution within the hydrogels, these results are extremely interesting from a procedural point of view. As far as we know, no results describing the tissue-like histological analysis of alginate-based hydrogels have been published right now. These experiments and the obtained data thus provide an additional tool for the analysis and characterization of alginate-based hydrogels for 3D cell cultures.

The composition of each type of hydrogel in terms of solid and liquid fractions is reported in Table 1. Recorded values are assimilable to the ones of the physiological hepatic microenvironment.^49,50^

**Table 1:** Solid and Liquid Fractions of ALG, GEL, and Hep3Gel^a^

hydrogel	solid fraction [%]	liquid fraction [%]
ALG	21.2 ± 1.5	70.8 ± 1.5
GEL	26.8 ± 5.6	63.2 ± 5.6
Hep3Gel	26.4 ± 2.1	63.6 ± 2.1

a Data are reported as mean ± SD.

Time-sweep tests allowed us to identify the gel point of each material, as reported in Table 2. This parameter indicates the moment at which materials start behaving as a solid rather than as a viscous solution. While for ALG and GEL, the values of this parameter almost coincide, for Hep3Gel, it is delayed by 60 min. Such behavior can also be appreciated from viscosity profiles, as reported in Figure S6. This can be explained by a combination of two phenomena connected to the preservation of GAGs. In the first place, these molecules are intrinsically able to buff pH variations; this also happens when lowering the pH of Hep3Gel precursor solution by adding the GDL, thus delaying the dissolution of CaCO_3_ and consequently crosslinking of Hep3Gel.^51^ Additionally, GAGs—especially heparin and heparan sulfate—are known to be able to bind Ca^2+^ ions, thus competing with alginate for them and interfering with crosslinking kinetics.^52^

**Table 2:** Gel Points of the Engineered Hydrogels

hydrogel	gel point [min]
Hep3Gel	120
GEL	60
ALG	60

**Figure 4:**
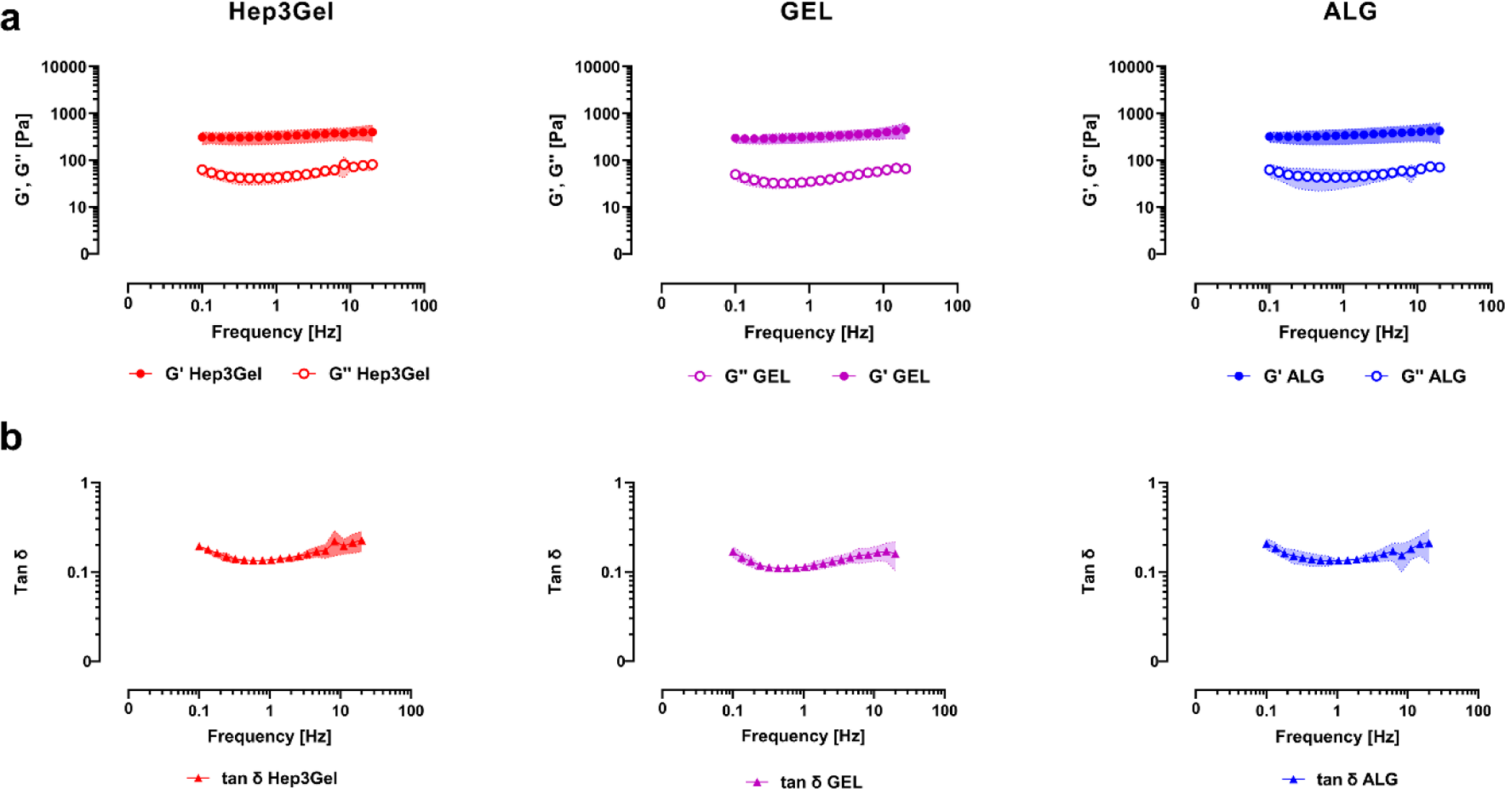
Rheological properties of the materials within their linear viscoelastic regions. (a) Conservative (*G*′) and loss (*G*″) components of the complex modulus of each kind of gel, measured as a function of the frequency. (b) Values of tan δ for each kind of hydrogel.

Frequency-sweep tests (Figure 4) showed that ALG, GEL, and Hep3Gel share similar values of *G*′, *G*″, and tan δ, denoting the solid-like behavior of the three types of hydrogels. The reaching of equal final viscoelastic properties reiterates that the pdECM buffer system only delays the dissolution of CaCO_3_, without limiting it. Moreover, the measured rheological parameters lie in the same range, characterizing the viscoelastic properties of a healthy murine model, thus mimicking the physiological hepatic biomechanical niche.^53^

These viscoelastic properties were obtained after optimizing the composition of Hep3Gel and control materials (Figure S7). The main idea driving the optimization of these parameters was that the mimicry of the physiological mechanical properties characterizing an organ is a primary requirement to be considered when designing and producing a three-dimensional matrix for cellular cultures.^54−56^ As a matter of fact, cells do not only respond to biochemical stimuli but also physical ones. The cytoskeleton can reshape as a function of the mechanical properties of the substrate, impacting in this way the cellular morphology and behavior.^57^ As a consequence, the matrix in which cells are embedded directly influences adhesion, proliferation, detachment, and differentiation pathways.^58−60^ Hence, mimicking these features of the physiological microenvironment is crucial for the maintenance of cellular phenotype, allowing in this way the expression of in vivo-like functionalities.

There are currently no published results measuring the viscoelastic properties of human or porcine livers using a rheometer. Some studies have measured the rheological properties of hepatic tissues from different species (including human beings and pigs) by the means of magnetic resonance elastography (MRE).^61−63^ These data show a similar value between the examined species, but it is still unclear whether it is possible to compare data from MRE with data from a rheometer that measures the viscoelastic response of samples to the applied shear.

However, it is commonly considered that human beings, swine, and rodents share an extremely high degree of similarity at the hepatic matrisome level.^64−69^ In light of these, together with the fact that—as a structural component—ECM is primarily responsible for the mechanical behavior of biological tissues, the viscoelastic properties of the developed hydrogels were tuned to match the ones of a physiological murine model, measured through rheometry.^53^

Additionally, all types of hydrogels resulted stable in culture conditions for up to 12 days. In this time window, no statistically significant weight variations—that may be directly related to the degradation of macromolecular chains—were recorded. The initial increase after the first 24 h in culture conditions is due to the swelling of hydrogels after being immersed in a fresh medium (Figure S8a).^70^ Frequency-sweep tests performed after 1 day in culture conditions highlighted for all materials a comparable decrease both in terms of *G*′ and *G*″ (Figure S8b). According to the literature, such a decrease is coherent with the swelling of hydrogels.^71,72^

Frequency-sweep analyses carried out in the subsequent days revealed no significant variations from data recorded after 1 day in culture (Figure S7b), thus recapitulating the stability of hydrogels in these conditions, during the whole experimental window. The same trend was observed when testing cell-laden hydrogels (Figure S7c). This behavior indicates that neither the proliferation of cells throughout the hydrogels nor their possible interactions with the hydrogel matrix is linked to a modification of the crosslinking meshes of Hep3Gel and control materials.

### 3.3 Characterization of the Shape-Shifting Behavior

Currently developed in vitro models of the liver are characterized by the use of various culture devices, with them being standard culture systems or tailor-made culture chambers. In this context, being a shape-shifter, intended as having the ability to be implemented within virtually any culture device, is a key feature of Hep3Gel. To this extent, the possibility of using Hep3Gel as flexibly as possible, producing both homogeneous self-spreading 3D matrices, and porous 3D-bioprinted structures, characterized by complex geometries, was considered a major requirement.

**Figure 5:**
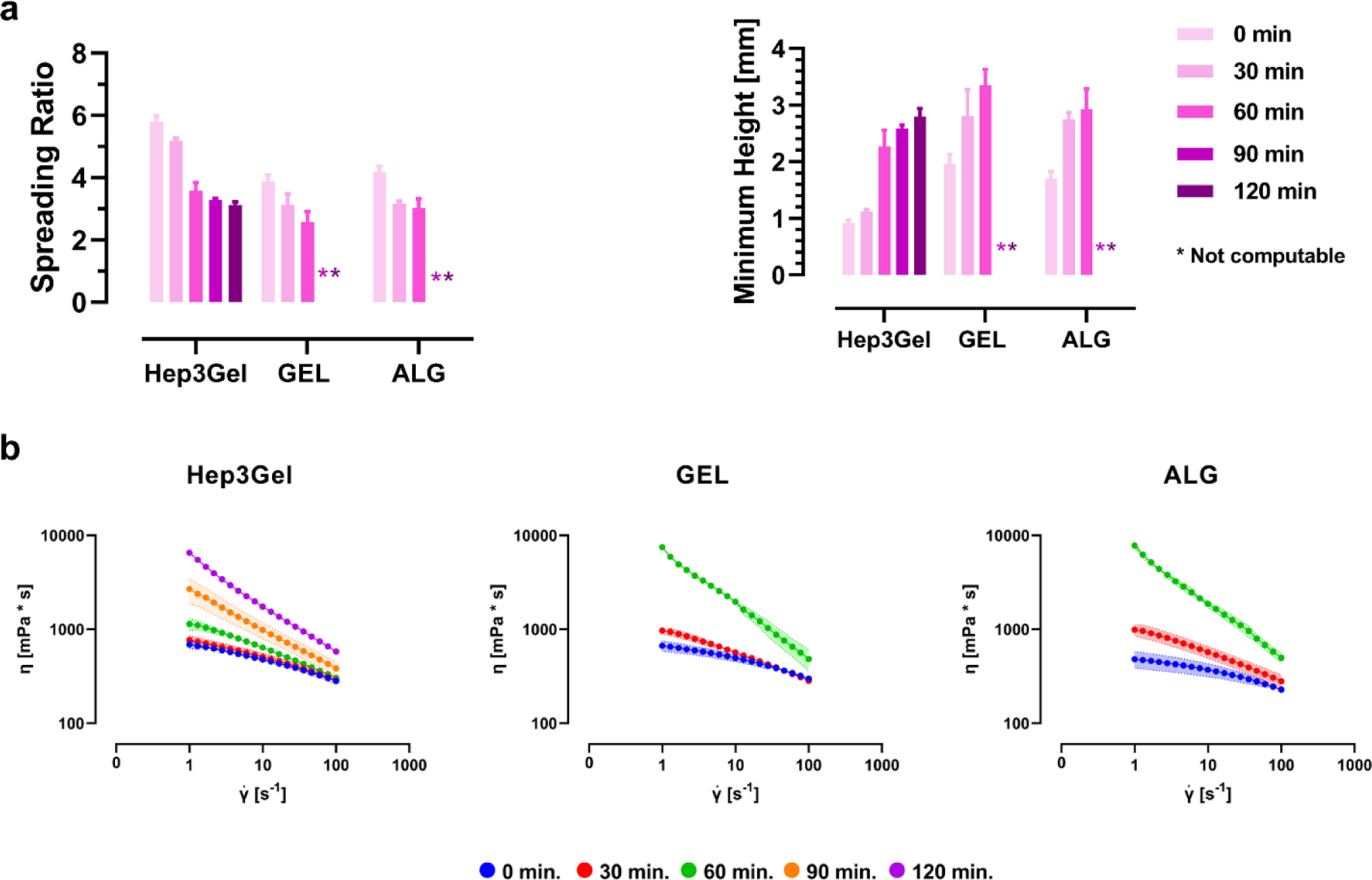
Characterization of the self-spreading behavior. (a) Spreading ratios and minimum heights of a 100 μL self-spreading drop of each material on a polystyrene flat surface at different time points between the beginning of crosslinking and the gel point. (b) Viscosity of Hep3Gel and control hydrogels expressed as functions of the shear rate  at different time points between the beginning of the crosslinking and the gel point.

Considering that, in the adopted experimental setup, volumes of drops were controlled with a micropipette, and in the absence of spreading, a drop of the fluid is characterized by a spherical shape, it was possible to calculate the diameter *D*_ideal_ of a nonspreading drop. Frames from the high-resolution videos of a drop spreading on a flat surface at different time points from the beginning of crosslinking (Figure S9), allowed the coherent computation of both the spreading ratios and the minimum heights of the layer of the hydrogel at the equilibrium. These parameters highlighted that the self-spreading ability of all the materials decreases with the advance of crosslinking (Figure 5a). In particular, Hep3Gel, GEL, and ALG displayed a self-spreading behavior up to 120, 60, and 60 min, respectively; these time points coincide with the gel points of hydrogels. As concerns the examined materials, it was noticed that after the gel point, it was not possible to characterize the self-spreading of drops since it was only possible to depose shapeless masses of gel rather than spherical drops.

The spreading of drops deposited on a flat surface is a direct way to characterize the ability of a fluid to uniformly wet a surface, thus adapting to the shape of a specific container.^73^ However, this is an extremely tricky phenomenon to study since both the spreading dynamics and the equilibrium conformations depend on a multitude of parameters related to the nature of both the substrate and the drop material, and mathematical models describing these phenomena in general conditions usually lead to complex numerical solutions.^74−76^ Since the described hydrogels have been specifically designed for tridimensional cell culture applications, this work is limited to the characterization of the self-spreading ability of the pregel solutions on polystyrene, which is the most common material to produce cell culture devices.

Spreading ratios greater than one imply the flowing of the material from the center of the drop itself to its peripheral region.^77^ According to Poiseuille’s law, the flow rate is inversely proportional to the viscosity η of the material, thus making η a crucial parameter to be considered when characterizing the spreading of a fluid.^78^ Viscosity curves of Hep3Gel, GEL, and ALG at the examined time points are reported in Figure 5b and highlight the shear thinning behavior of hydrogels. Moreover, sequential measurements underline the progressive increase of viscosity, thus explaining the decrease of *S*_0_ through time.

Finally, hydrogels extruded prior to the gel point into different types of multiwell plates showed the ability to homogeneously cover the bottom of the well, perfectly adhering to it independently from the radius, without forming any bubble or hole (Figure S10a,b). When hydrogels were extracted from the wells after their crosslinking, no structural damages were observed; moreover, the characteristic dimensions and shapes imposed by the wells were retained (Figure S10c).

This qualitative result reiterates the possibility of producing a uniform and homogeneous self-spreading matrices with the desired shape, which can be easily extracted from their molds and handled for further operations, including the production of 3D cell cultures.

The use of homogeneously structured 3D matrices for culturing cells has already been validated, demonstrating their suitability to sustain cellular cultures through time while ensuring the beneficial effects which are typical of a three-dimensional culture.^18^ However, there are some cases, usually dependent on the shape and the dimension of the construct, as well as on the culture conditions, in which 3D bioprinting is crucial to manufacturing macroporous structures, aiming, for instance, to homogenize the diffusion of oxygen and nutrients throughout the construct or to allow the uniform flow of the medium in dynamic cultures.^79−81^

Considering this, Hep3Gel was designed aiming to also offer the possibility to be used as bioink, leaving in this way to the final users the wider possible flexibility of use.

**Figure 6:**
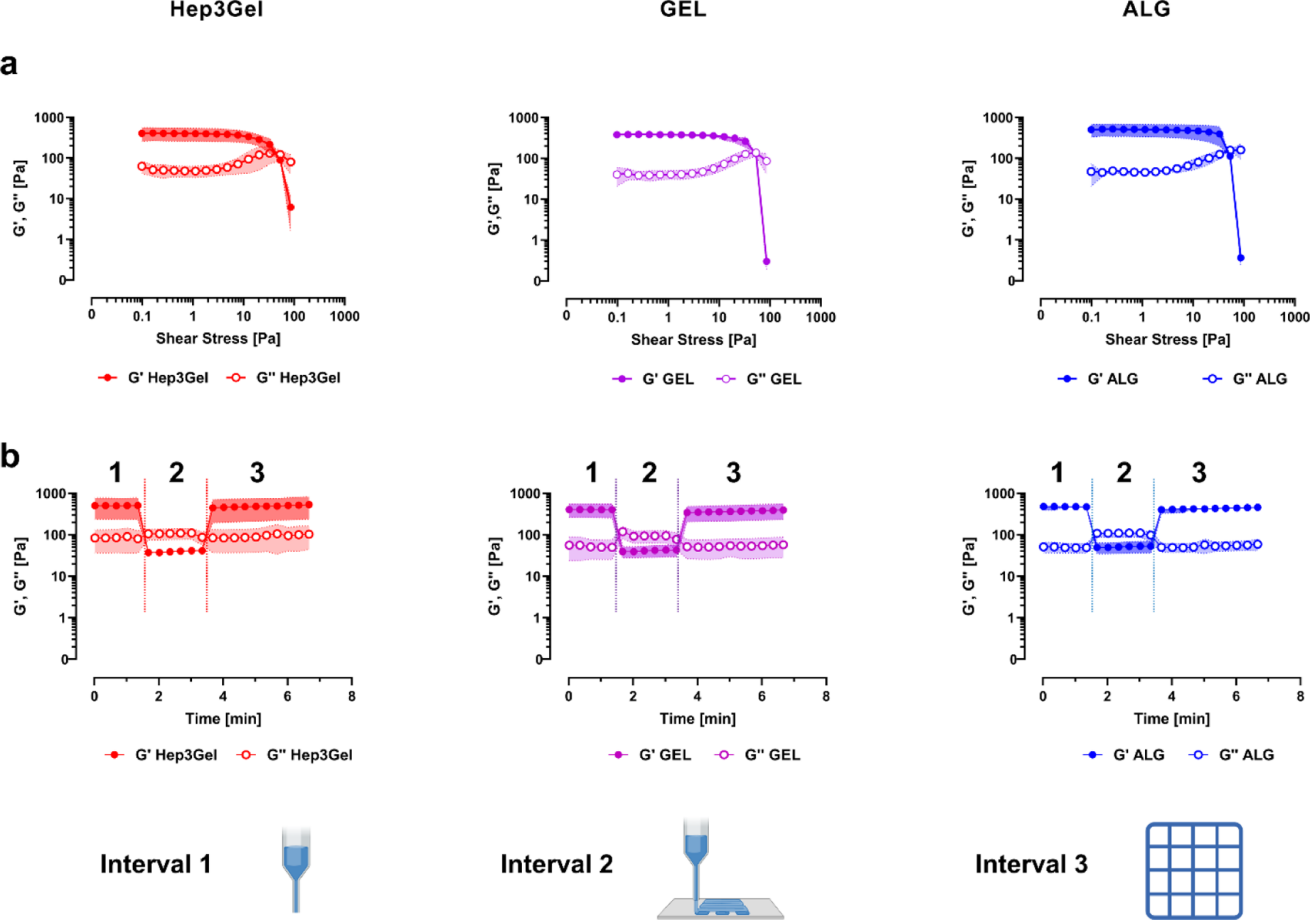
Rheological tests to theoretically assess the possibility of extruding ALG, GEL, and Hep3Gel from a syringe. These tests aim to provide a priori a proxy of the possibility to process materials through extrusion-based 3D-bioprinting. (a) Refers to the flow test and simulates the extrusion in correspondence with different shear stresses. The presence of a yielding point is highlighted by *G*″ starting prevailing over *G*′. (b) Reports the results of the recovery test, showing that all of the hydrogels can recover their original viscoelastic properties after being extruded. In particular, the first interval aims to mimic the bioink in the cartridge before being printed. The second interval is designed to mimic the extrusion procedure, through a 100% shear strain. Finally, the third interval mimics the construct after the printing at the equilibrium.

A priori, specific rheological analyses highlighted the possibility to extrude Hep3Gel and control materials without impairing their properties (Figure 6). In particular, during the first test, the possibility of having the hydrogel flowing during the extrusion process was checked. The presence of yielding points, defined as the point at which *G*″ starts to prevail over *G*′, in Hep3Gel, GEL, and ALG implies the existence of a shear stress level at which the hydrogels start behaving like viscous fluids, being thus able to be extruded.^31,82^ The second test was designed to include three different time windows investigating the properties of gels before yielding, when yielded, and after the yielding, respectively. With this test, it was possible to appreciate that despite changes in their rheological parameters due to the yielding occurring during extrusion, all the hydrogels were able to recover their original properties, thus highlighting the existence of some conditions in which the hydrogels are 3D printable.^31,82^

The a priori knowledge of the yielding point of a bioink is crucial to reduce the trial-and-error procedure typical when studying the printability of a material. Actually, under the hypothesis that syringe extrusion is a process in which a high flow rate—associable with high-frequency deformations—dominates, the extrusion pressure needed to produce specific shear stresses inside the syringe can be estimated through a first-order mathematical model, as reported in eq 7.^83^
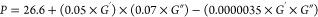
7

In this way, the minimum extrusion pressures have been theoretically quantified for all of the hydrogels in correspondence to yielding points and are listed in Table 3, together with the minimum pressure at which a uniform filament of the material was extruded by the 3D bioprinter and with the pressures needed to extrude a continuous fiber, as experimentally determined.

**Table 3:** Yielding Points and Estimated Printing Extrusion Pressures of Hep3Gel, GEL, and ALG

hydrogel	yielding point (*G*′ = *G*″) [Pa]	minimum extrusion pressure (model) [kPa]	minimum extrusion pressure (experimental) [kPa]	fiber printing pressure [kPa]
Hep3Gel	205	52	43	56
GEL	155	44	48	59
ALG	121	41	45	57

According to the literature, the identified extrusion pressures reside in the best range possible, allowing optimal extrudability without impairing cellular viability.^83^

**Figure 7:**
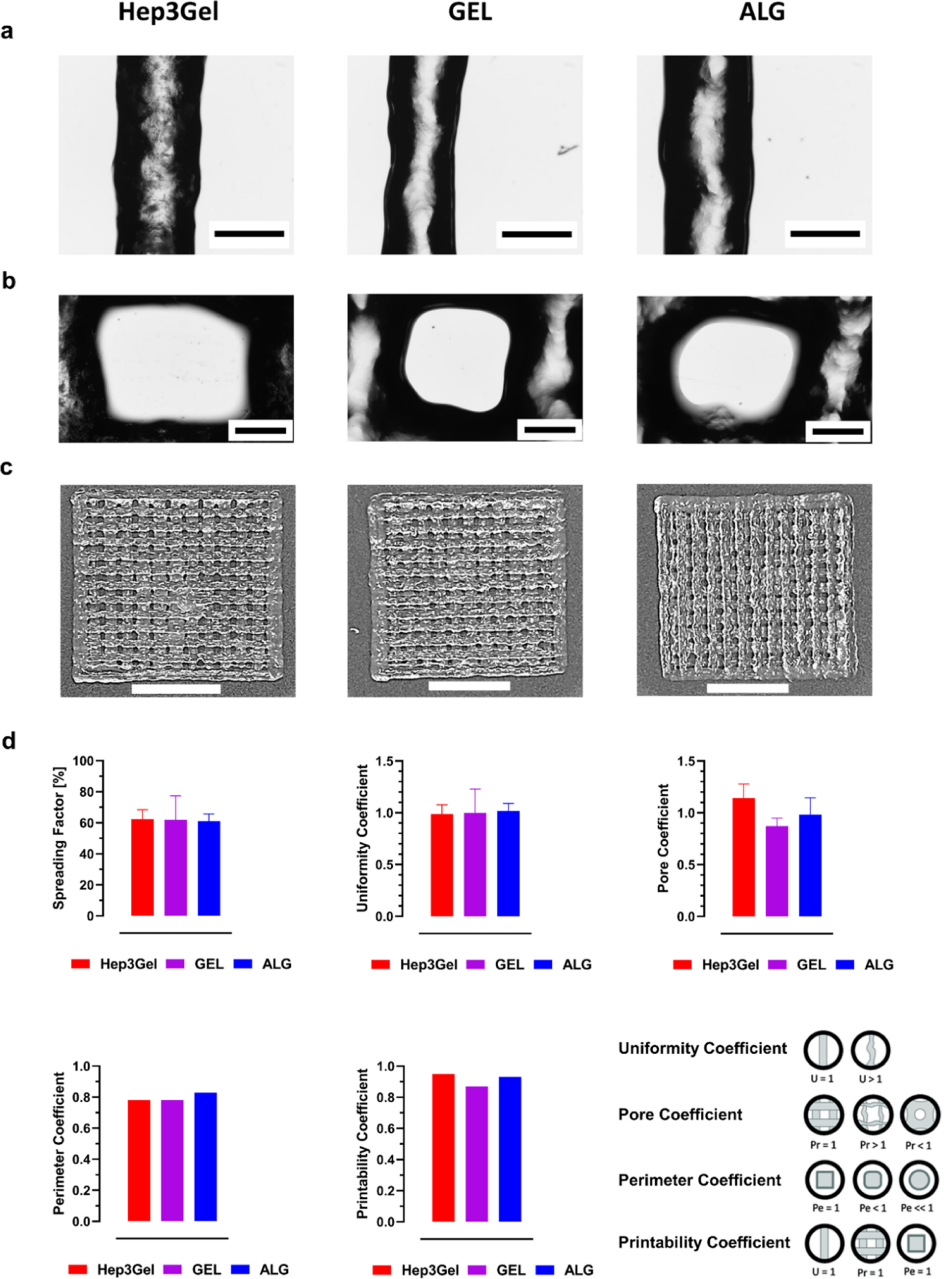
Printability of Hep3Gel and control materials. (a) Details of extruded fibers; the scale bars correspond to 500 μm. (b) Details of the printed pores and the scale bars correspond to 500 μm. (c) Overall pictures of printed grids; the scale bars correspond to 1 cm. (d) Shape fidelity and printability parameters. Since standard deviations are already considered during the computation, error bars are displayed neither in the graph of the perimeter coefficient nor in that of the printability coefficient. The insets illustrate the conformations of ideal and nonideal geometries.

The outcomes of the printing procedure, implementing the fiber printing pressures, as reported in Table 3, are shown in Figure 7.

*S*, the spreading factor, depends on the speed of the extruder and the extrusion pressure; it is expressed as a percentage and quantifies the relaxation of the fiber after the extrusion. Conversely, for all the other computed coefficients, the unit value represents the optimal condition, in which the maximum shape fidelity—intended as the degree of correspondence between the CAD design and the 3D-printed geometry—is achieved. Qualitative observations of the printed structures, together with the slight displacement from the optimal value displayed by *U*, *P*_r_, and *P*_e_ suggest that all the materials are suitable to be manufactured with EBB techniques. Regarding the printability coefficient instead, *P* is a recently developed parameter aiming to combine in a single computation the information obtained by the average values of the previous parameters.^31^ Also, for *P*, the optimal value is represented by the unit and can be reached when the printed structure is perfectly adherent to the CAD geometry in terms of the perimeter (*P*_e_ = 1), well-structured and superimposed 3D layers (*P*_r_ = 1), composed of straight fibers (*U* = 1).

These results, combined with the ones reported in Figure 6 showing the extrudability of materials and their ability to recover their original viscoelastic properties after the extrusion, indicate that the hydrogels are suitable to be manufactured with EBB, with slight displacements (<−10% in the case of the Hep3Gel printability coefficient, Figure 7d) from the designed CAD geometry.

It must also be considered that printability is not a property of the material but rather a condition affected not only by multiple properties and parameters but also by the to-be-printed geometry and by the properties of the substrate on which the material has to be printed.^84^ In this study, it was possible to print the considered materials following the designed geometry by tailoring their printability through the ad hoc setting of the printing parameters.

Additionally, when working with alginate-based hydrogels, the printability and its tuning also depend on the crosslinking strategy, be it an external or an internal gelation.^85^ Externally crosslinking alginate hydrogels is the most adopted solution to print this material. This technique is based on extruding alginate gel precursors in a solution with directly available Ca^2+^ ions or other divalent cations, producing immediate gelation. Therefore, optimizing the printability when exploiting this strategy relies on adjusting the chemistry of the materials and the printing parameters as a function of the specific application.^86,87^ In this work, we adopted a less common strategy, by internally crosslinking alginate. These hydrogels are produced by mixing the precursor solution with a suspension in which Ca^2+^ ions are not immediately available but can be set free in a subsequent moment by adding specific acidic reagents. This crosslinking technique was preferred to external gelation due to the possibility to obtain a homogeneous degree of crosslinking throughout the structure of the hydrogels, thus offering the possibility of finely controlling the final viscoelastic properties of the materials.^88^ Conversely, external gelation leads to an inhomogeneous degree of crosslinking, characterized by a “core–shell” structure, in which the outer surface has a different crosslinking degree than the inner part. This structure affects the viscoelastic properties of the materials and their control.^89,90^ Moreover, with internal crosslinking, the speed at which divalent cations are released, and thus the crosslinking kinetics, can be controlled by varying the concentration of the acidic reagent, providing in this way the possibility of optimizing the processability of the material as a function of the time, with an approach known as reactive bioprinting.^31^

**Figure 8:**
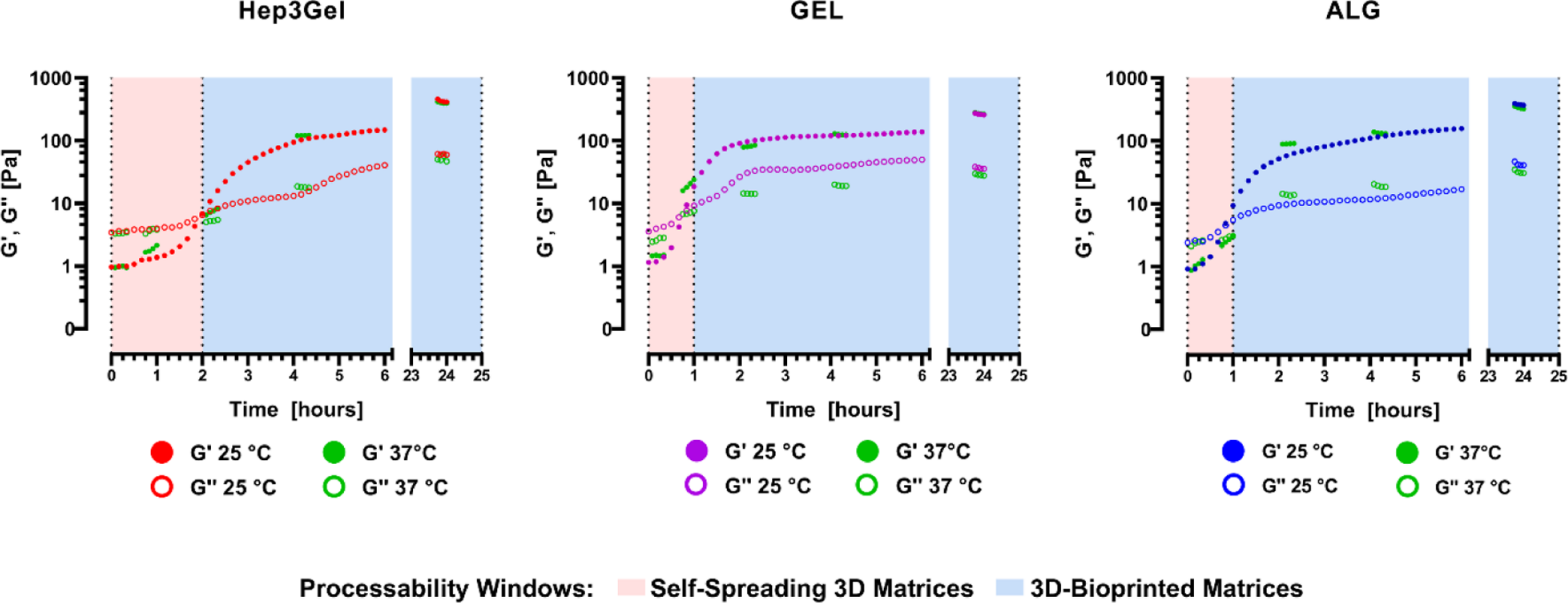
Crosslinking kinetics of Hep3Gel and control materials. Results were obtained through time-sweep analyses, both at RT and 37 °C. Processability windows during which exploiting the materials to produce self-spreading or bioprinted three-dimensional matrices are highlighted for each material.

In light of this, the compositions of Hep3Gel, GEL, and ALG have been finely tailored to display slow crosslinking kinetics (Figure 8).

Temperatures in the considered range display no impact on the crosslinking kinetics of each hydrogel, making them suitable to manufacture the same construct at different temperatures and overcoming in this way a strict boundary imposed by a wide range of materials characterized by thermoresponsive features.^91,92^

The tailored reaction kinetics leave a suitable room of maneuver for further operations, not only when optimizing the hydrogels for specific EBB applications but also in case of exploiting them for the production of homogeneous, self-spreading structures. Furthermore, in detail, the slow progression of the gelation through time allowed us to identify for each of these hydrogels two different processability windows, characterized by deeply different rheological properties. When in the first window, materials are characterized by their viscous component prevailing on the elastic one, thus being able to spread and adapt their shape to that of the desired molds. Conversely, after the gel point, the elastic nature of materials starts to prevail over the viscous one, making them able to retain the shape imposed during the printing.

To sum up, we avoided producing two different alginate-based hydrogels, one for self-spreading matrices and one for the bioink as the very same formulation allows us to fabricate a single hydrogel with a dual behavior. This dual behavior that determines the shape-shifting nature of the engineered materials might be potentially achieved with other materials also whose crosslinking kinetics are tunable as a function of the time and that can be homogenously crosslinked throughout their bulk.

The presented results represent a proof of concept of the suitability of Hep3Gel and control materials to be processed via EBB and lay the foundations to explore further printing scenarios, each of them requiring its own specific tuning.

### 3.4 Cell Distribution and Viability Analyses

**Figure 9:**
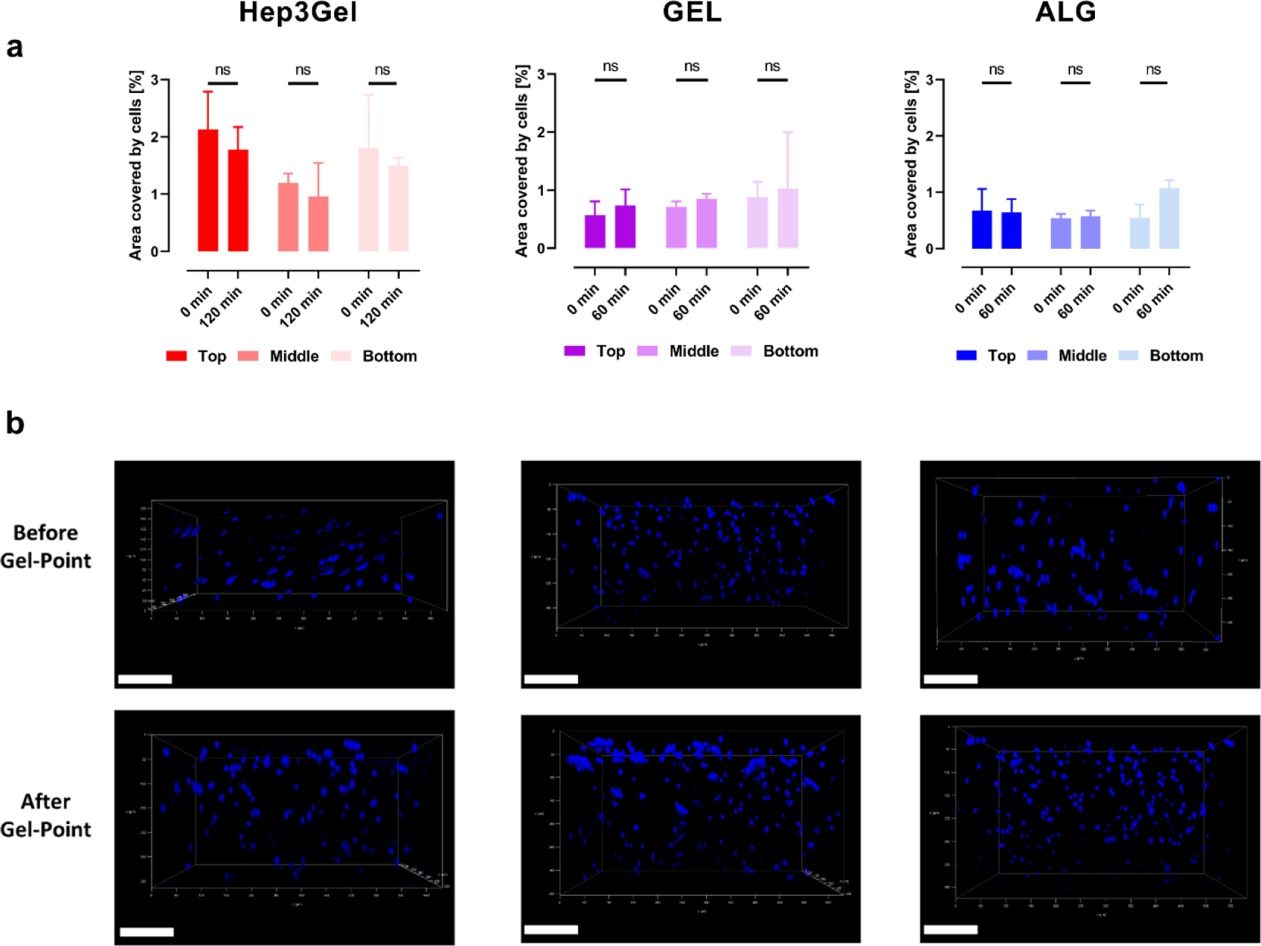
Spatial distribution of cells before and after the gel point. (a) Quantitative measurements of the area covered by nuclei of cells in Hep3Gel and control materials at the top, middle, and bottom layers. Measurements were performed on images acquired before and after the gel point, respectively. (b) View of the cellular distribution through the vertical cross sections of Hep3Gel and control materials, before and after the gel point. The nuclei of cells are stained in blue with Hoechst 3342. The scale bars correspond to 100 μm.

The quantification of the area covered by cells in CLSM images from the top, middle, and bottom planes of Hep3Gel and control materials revealed no statically significant differences between the same plane at the beginning of the crosslinking and immediately after the gel point. Moreover, no statistically significant differences were recorded between the homologous planes of different materials (Figure 9a). A comprehensive overview of cellular distribution within hydrogels throughout their vertical cross section can also be appreciated in Figure 9b. The absence of quantitatively relevant differences between planes before the gel point shows that cells are homogeneously dispersed throughout the volume of the materials. Additionally, the preservation of this situation after the gel point demonstrates that, during the examined time window, no precipitation of cells nor their aggregation is induced, as shown in Figure S11.

**Figure 10:**
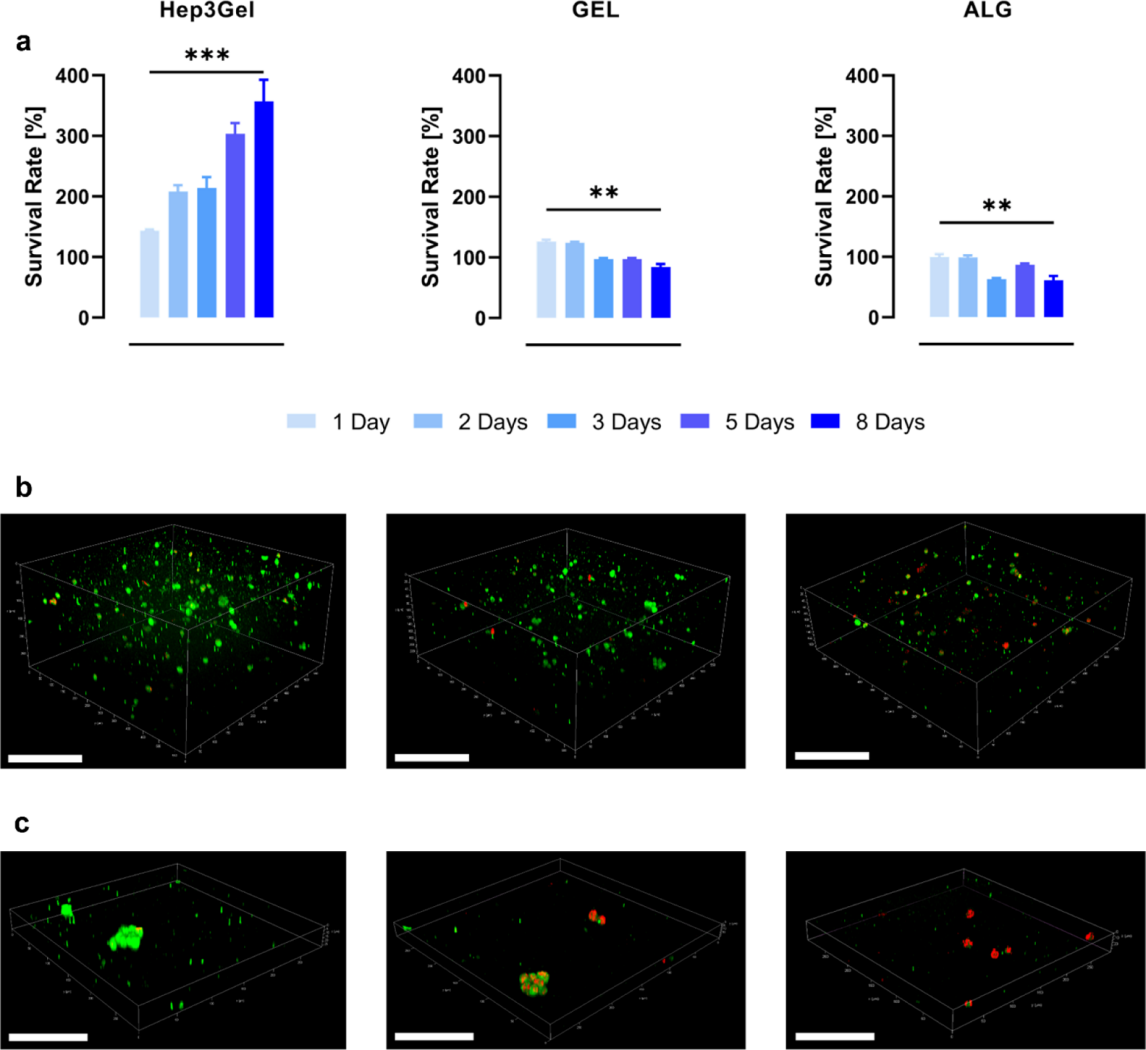
Viability analyses on 8 day cell cultures. (a) Quantitative analyses of viability on each type of hydrogel at different time points; data were normalized as a percentage of the viability measured 4 h after the encapsulation. (b) CLSM qualitative viability analyses performed using a LIVE/DEAD viability/cytotoxicity kit as reported in the Experimental Section. All CLSM images refer to the core regions of hydrogels after 8 days in static culture. Green spots indicate the presence of live cells, while red and yellowish spots refer to dead or suffering cells. The scale bar corresponds to 100 μm. (c) 40× magnification of the core region of Hep3Gel after 8 days in a culture that highlights the presence of viable in vivo-like cell aggregates. The scale bar corresponds to 50 μm.

Cell viability was evaluated through a combination of quantitative and qualitative techniques. Quantitative measurements of the metabolic activity (Figure 10a) highlighted a significant increase in the viability of Hep3Gel-cultured cells from day 2 to day 8. Conversely, the viability of cells cultured within both ALG and GEL follows approximatively the same trend—characterized by a significant decrease after day 3—during the whole experimental window. The presence of the ECM in Hep3Gel favors cell adhesion and growth, increasing the cell viability by almost 400% during the considered time points. Live/dead fluorescence CLSM observations of the core regions of gels after 8 days in culture (Figure 10b) confirmed the quantitative results obtained with the MTT assay and revealed the homogeneous distribution of cells throughout the hydrogels. The maintenance of an almost fully viable core within a three-dimensional cellularized construct usually represents a major challenge.^93,94^ This obstacle is even exacerbated when 3D matrices are cultured in static conditions due to the lack of fluxes forcing medium renewal in the inner districts of a cellularized construct.^95^

Moreover, CLSM observations showed that in both GEL and ALG, there is a higher density of dead (stained in red) or suffering cells (yellowish color). Interestingly, Hep3Gel was not only characterized by a higher overall cellular density but also by a significantly higher survival rate since the most of cells in Hep3Gel were only stained with the green dye. This result reiterates the extremely positive impact of pdECM powder on seeded cells. Additionally, we hypothesized that pdECM particles do not act only as a secure outpost, providing cells with in vivo-like interaction sites, but they also ease the permeation of oxygen and nutrients throughout the whole structure of Hep3Gel, enhancing in this way the viability even in the core regions of the matrix.

Finely mimicking the in vivo chemomechanical environment is crucial to study cellular behavior in a physiological-like environment. Our results indicate that fully viable cellular aggregates can be spotted in Hep3Gel as observed with CLSM (Figure 10c). These clusters were appreciated after 8 days of culture and are extremely similar to the ones formed in vivo by HepG2 cells.^96^ Their presence was only reported in Hep3Gel, and no viable clusters were observed either in GEL or in ALG hydrogels.

## 4 Conclusions

Different well-established decellularization strategies have been combined to set up a tailored procedure, able to preserve the integrity of structural and functional components of the ECM. Porcine liver ECM powder embedded within an alginate hydrogel allows for achieving rheological properties which are compatible with the ones of the natural tissue. Hep3Gel has been demonstrated to be able to enhance cellular proliferation in the medium term, even promoting the formation of in vivo-like aggregates, which were not observed in control hydrogels. The ability of Hep3Gel to adapt to the shape of virtually any culture device, combined with the possibility to 3D-bioprint it, shows the versatility of this material in the production of tridimensional in vitro models of the liver. This hybrid hydrogel thus provides a novel additional tool that can be applied to unveil the complexity of hepatic physiology.

This work has to be considered as a pilot study of the feasibility to mimic the hepatic chemomechanical environment with Hep3Gel and to use it to fabricate cellularized 3D matrices with different manufacturing techniques. In light of this, many aspects should be still investigated, aiming to validate Hep3Gel-based in vitro models of the liver. In the first place, the possibility of culture and coculture of different hepatic cell types has to be assessed. Additionally, due to the nature of this behavior, shape-shifting hydrogels can be produced with alginate and potentially with those polymers whose crosslinking is induced by ionic mechanisms, thus allowing tuning kinetics as a function of time.

## Supporting Information Available

Steps to prepare Hep3Gel, GEL, and ALG; optical microscopy of the pdECM powder; SDS-PAGE of type I collagen and fibronectin; dot-blot quantification of collagen and fibronectin; details of immunohistochemical images; viscosity profiles of Hep3Gel and control materials during the crosslinking; different formulations of Hep3Gel and control materials, tested during the optimization phase; stability of materials in culture conditions up to 12 days in terms of weight variation and time-dependent rheological analyses; drop deposition and spreading; self-spreading matrices produces in different multiwell sizes; and cellular distribution within Hep3Gel, GEL, and ALG before and after the gel point (PDF)

## Abbreviations

ECM: extracellular matrix
pdECM: porcine liver decellularized ECM
RT: room temperature
PD: samples of pdECM powder
PN: samples of native porcine liver
Coll I: type I collagen
Fn: fibronectin
GAG: glycosaminoglycan
FBS: fetal bovine serum
ALG: only alginate-based hydrogel
GEL: alginate and gelatin-based hydrogel
*S*_0_: spreading ratio
*S*: spreading factor
*U*: uniformity factor
*P*_e_: perimeter coefficient
*P*_r_: pore coefficient
*P*: printability coefficient
CLSM: confocal laser scanning microscopy
EBB: extrusion-based bioprinting
