# Supplementary material for: Hep3Gel: A Shape-Shifting Extracellular Matrix-Based, Three-Dimensional Liver Model Adaptable to Different Culture Systems
Source: ACS Biomater Sci Eng. 2022 Dec 16;9(1):211–29. doi: 10.1021/acsbiomaterials.2c01226 (PMC9832437; doi:10.1021/acsbiomaterials.2c01226)
Supplement: Supplementary file 1 — ab2c01226_si_001.pdf [file ab2c01226_si_001.pdf]

SUPPORTING INFORMATIONS

Hep3Gel: a shapeshifting extracellular matrix-based, three-dimensional liver model adaptable to different culture systems

Giuseppe Guagliano [a], Cristina Volpini [b], Lorenzo Sardelli [a], Nora Bloise [b], Francesco Briatico-Vangosa [a], Antonia Icaro Cornaglia [c], Silvia Dotti [d], Riccardo Villa [d], Livia Visai [b,e,f] and Paola Petrini* [a,g]

* Email: Prof. Paola Petrini

paola.petrini@polimi.it

a. Department of Chemistry, Materials, and Chemical Engineering "G. Natta", Politecnico di Milano, Piazza Leonardo da Vinci 32, 20133 Milan, Italy

b. Molecular Medicine Department (DMM), Center for Health Technologies (CHT), UdR INSTM, University of Pavia, Pavia, Italy.

c. University of Pavia - Department of Public Health, Experimental and Forensic Medicine, Histology and Embryology Unit, Pavia, Italy

d. National Reference Center for Alternative Methods, Welfare and Care of Laboratory Animals, Istituto Zooprofilattico Sperimentale della Lomabardia ed Emilia Romagna, Brescia, Italy

e. Medicina Clinica-Specialistica, UOR5 Laboratorio Di Nanotecnologie, ICS Maugeri, IRCCS, Pavia, Via Boezio, 28-27100, Pavia, Italy

f. Interuniversity Center for the promotion of the 3Rs principles in teaching and research (Centro 3R), Università di Pavia Unit, Italy

g. Interuniversity Center for the promotion of the 3Rs principles in teaching and research (Centro 3R), Politecnico di Milano Unit, Italy

Supplementary Figure 1

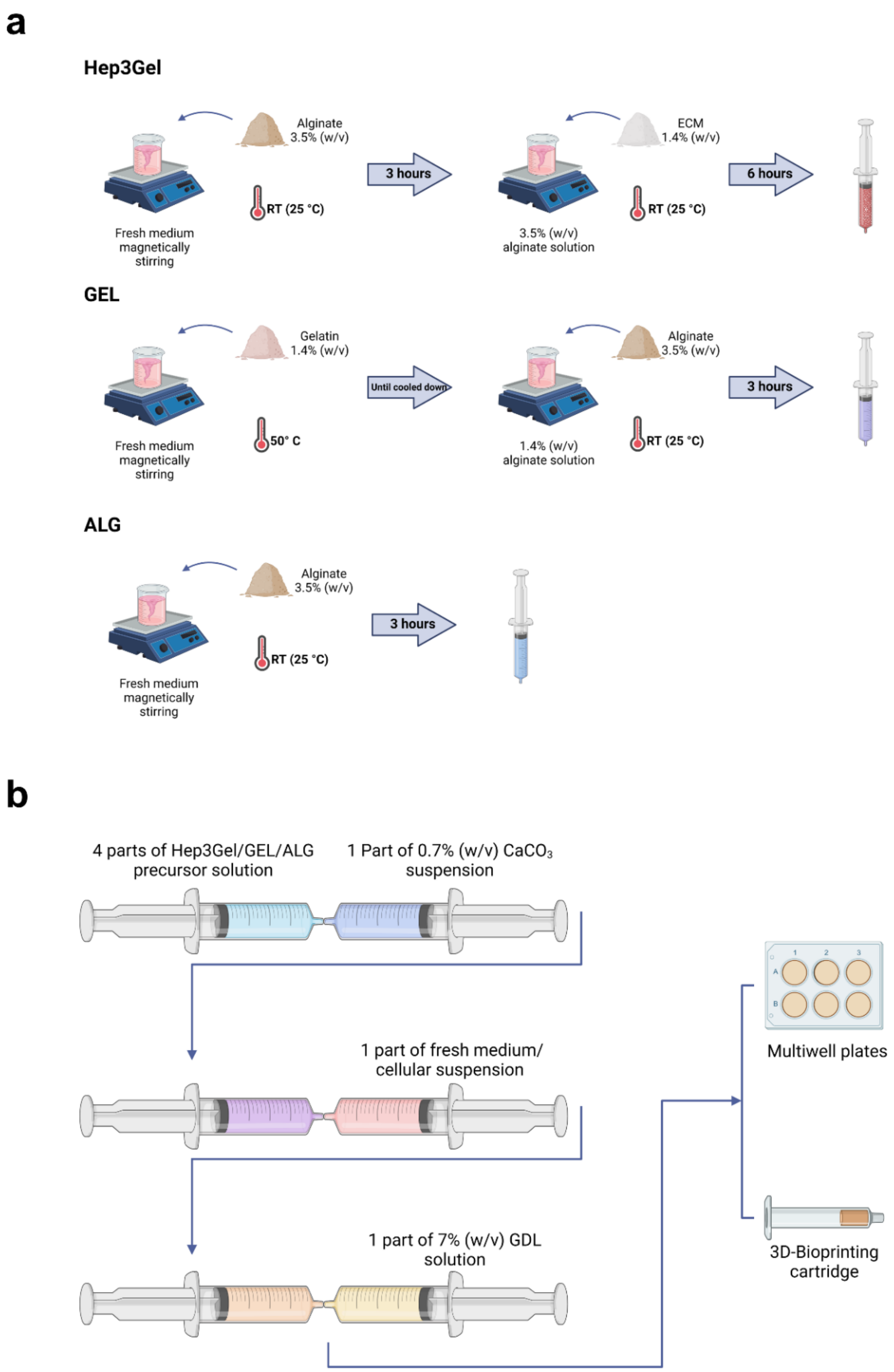

Figure S1 Procedural step to produce Hep3Gel and control materials. a) Preparation of the hydrogel precursor solutions of Hep3Gel, GEL, and ALG. b) Sequential mixing of the precursor solutions with calcium carbonate, fresh medium/cells suspension, and GDL, thus inducing the crosslinking, and final extrusion in a multiwell plate or in a bioprinting cartridge, depending on the needs.

Supplementary Figure 2

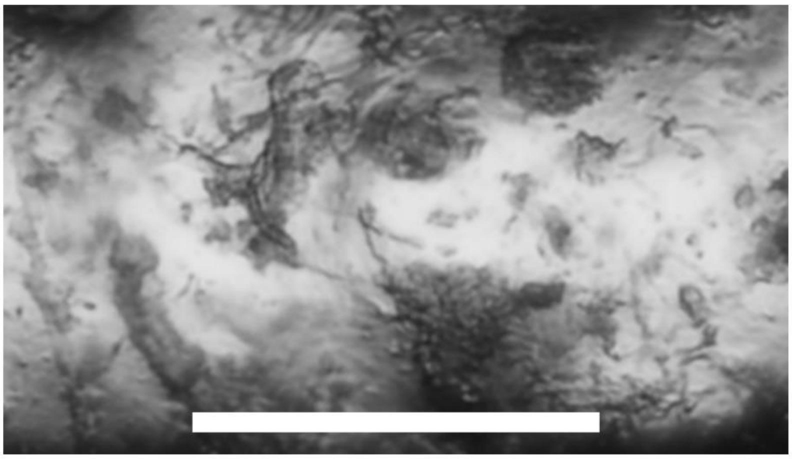

Figure S2 Optical microscopy image of pdECM fibrillar powder. Suspension of pdECM observed with an optical microscope, 5X magnification. pdECM powder displays a fibrillar irregular shape, its dimensions were measured along different directions with FIJI. The scalebar corresponds to 250 µm

Supplementary Figure 3

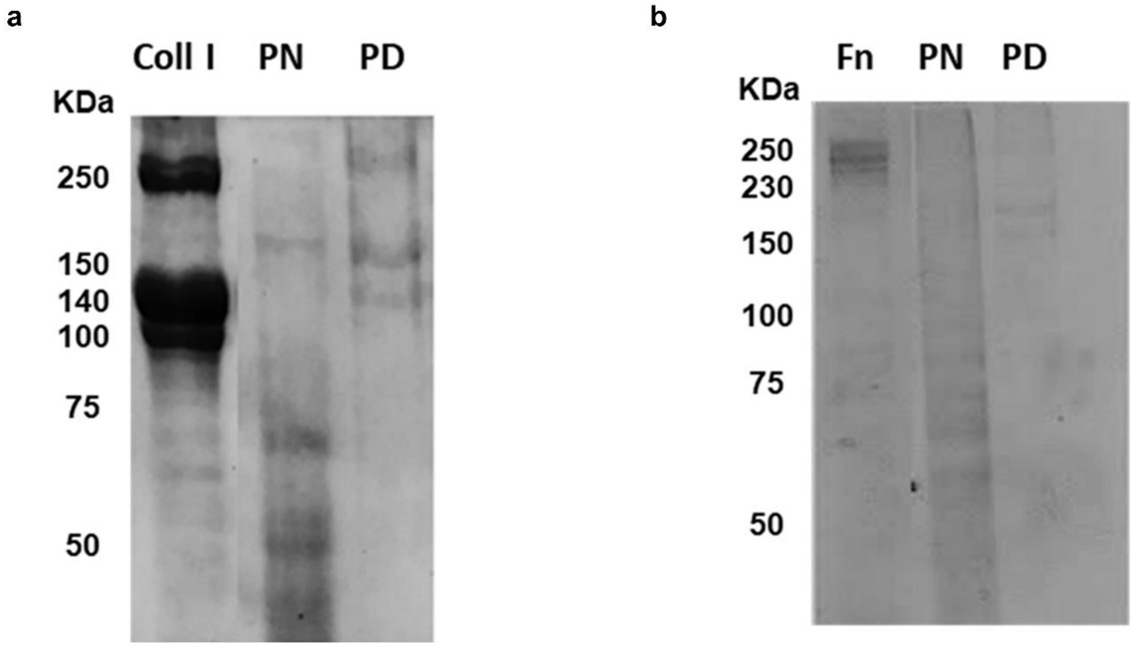

Figure S3 Collagen I and fibronectin content by SDS-PAGE a) A representative image of SDS-PAGE gel electrophoresis performed in non-reducing conditions b) A representative images of SDS-PAGE gel electrophoresis performed in reducing conditions. Coll I, Fn, PN, PD refer to type I collagen, fibronectin, native porcine sample, and decellularized porcine sample, respectively

Supplementary Figure 4

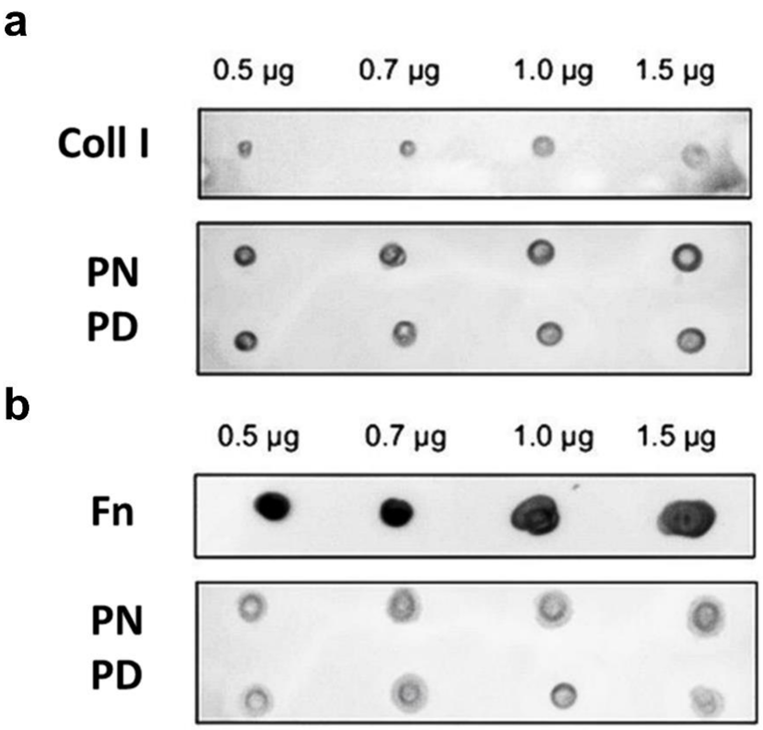

Figure S4 Dot blot for type 1 collagen and fibronectin. Sample were spotted onto nitrocellulose membranes at different concentrations (0.5µg – 1.5 µg) and overlaid with a) anti-type I human collagen and b) anti-human fibronectin antibody, respectively

Supplementary Figure 5

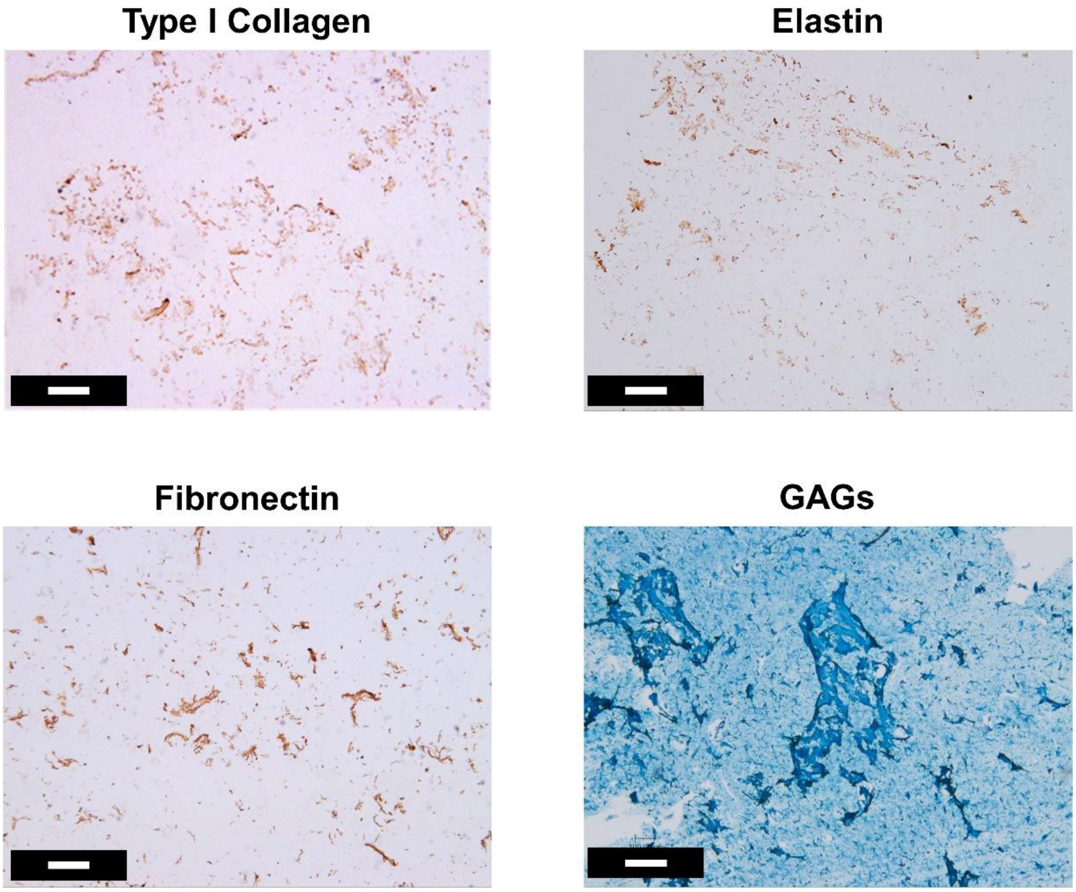

Figure S5 Imaging from immunohistochemical analyses. Example of the single images of type I collagen, elastin, fibronectin, and GAGs acquired at a 4X magnification. Different images of these type were acquired from contiguous fields on the sample, and then digitally combined, aiming to provide the overall view of the whole field. The scale bars correspond to 200 µm.

Supplementary Figure 6

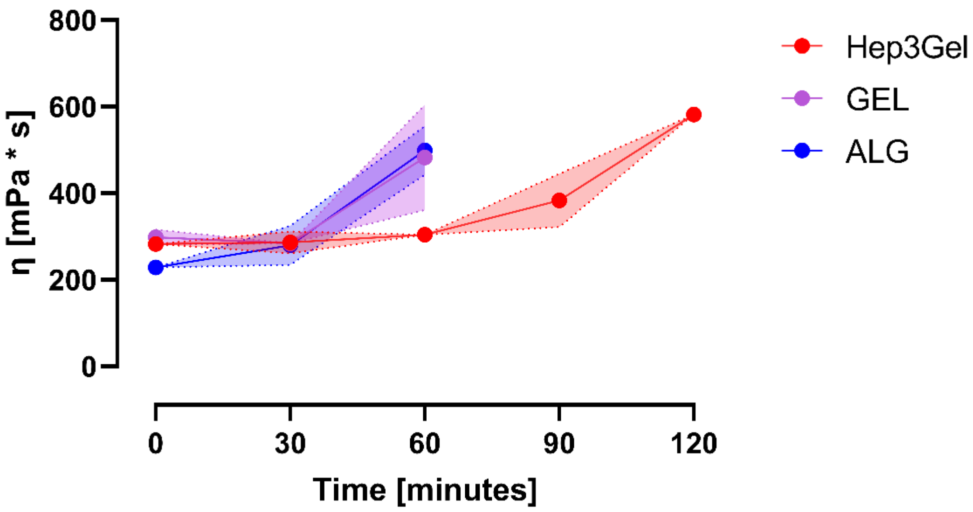

Figure S6 Viscosity profiles of Hep3Gel and control materials. Viscosity profiles of hydrogels measured every 30 minutes from the beginning of crosslinking up to the gel-point. Measurements were carried out at a constant shear-rate $\dot{\gamma}$, equal to 100 s^{-1}

Supplementary Figure 7

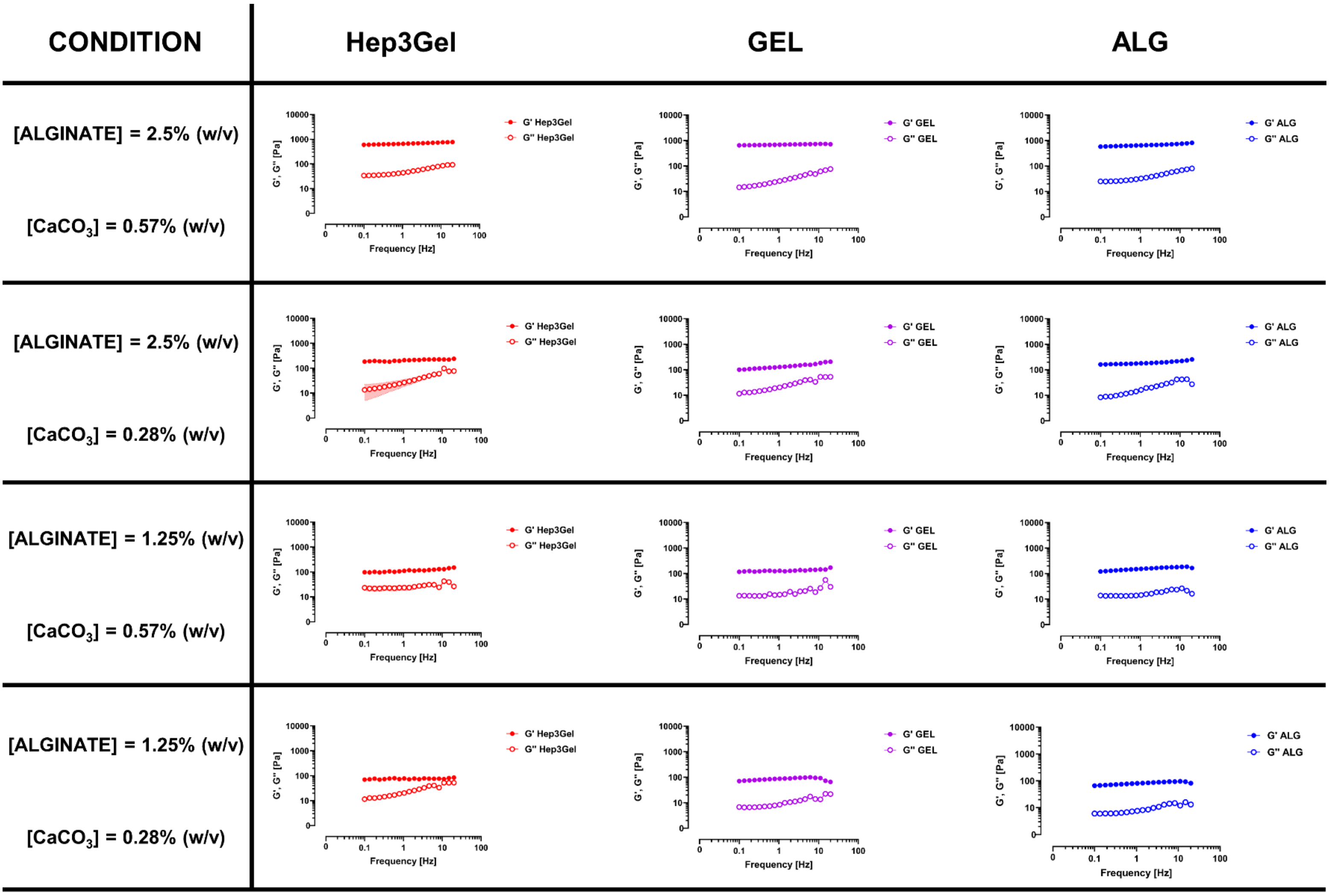

Figure S7 Optimizing the formulation of Hep3Gel and control materials. To optimize the formulation of the hydrogels, the rheological behavior of the material was characterized implementing different concentrations of alginate and calcium carbonate. The optimal formulation was determined as the one that better mimicked the viscoelastic properties of a physiological murine organ.

Supplementary Figure 8

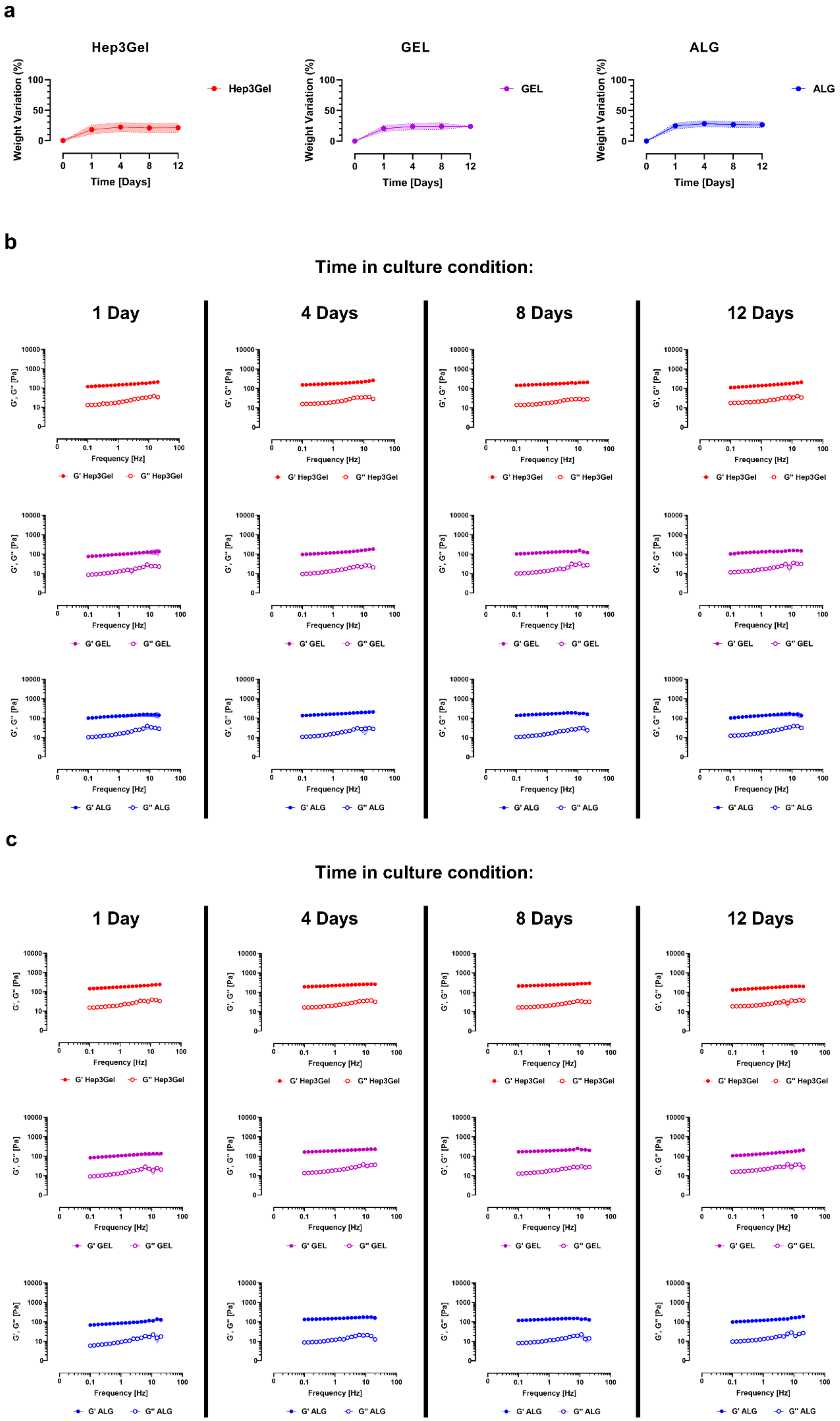

Figure S8 Stability of Hep3Gel and control materials in culture conditions. The stability of hydrogels was studied by keeping them in culture conditions up to 12 days after the end of crosslinking. a) After the initial weight increase, that is coherent with the swelling of hydrogels, no significant weight variations are recorded during the experimental time-course, indicating no degradation of the macromolecular chains. Rheological analyses carried out at homologous time point highlighted a decrease of the viscoelastic properties of both cell-free (b) and cell-laden (c) hydrogels after 1 day in culture conditions, that is coherent with their swelling. No further variations of the viscoelastic properties were recorded during the experimental time-window for cell-free (b) and cell-laden (c) hydrogels.

Supplementary Figure 9

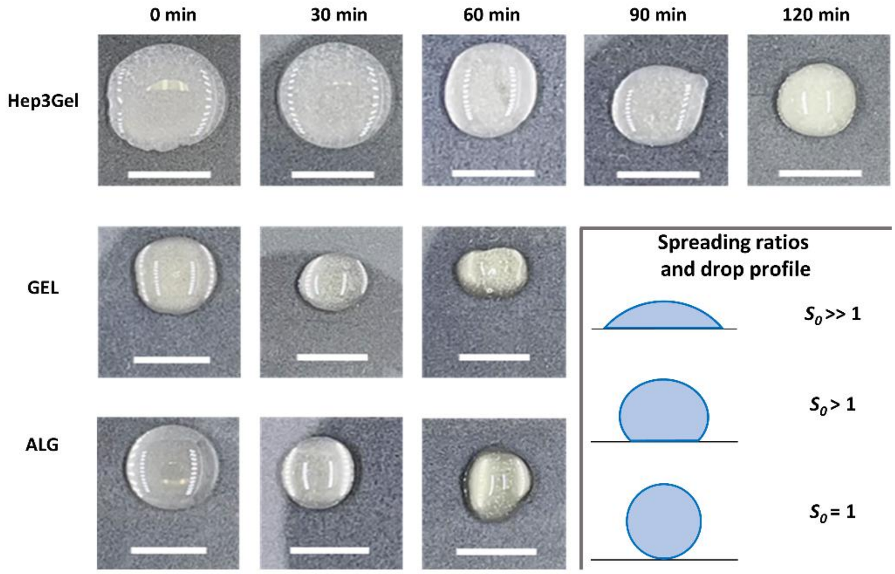

Figure S9 Drop deposition and spreading. Drops of Hep3Gel, GEL, and ALG were deposed on a flat polystyrene surface at different time-points between the beginning of the crosslinking and the gel-point. Frames highlight the decrease in self-spreading as the reaction progresses. Scalebars correspond to 1 cm. The bottom right corner of the picture qualitatively illustrates how the equilibrium profile of a deposed drop varies as function of the spreading ratio S_0

Supplementary Figure 10

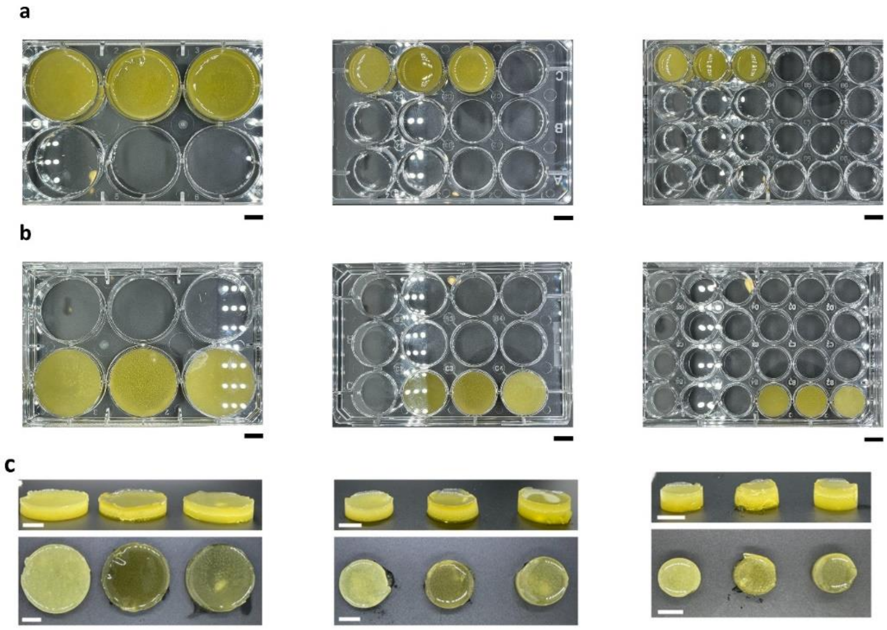

Figure S10 Self-spreading matrices adapted their shapes to different multi-well sizes. a) and b) report the top and bottom views of the multi-wells, respectively. Hydrogels covered the wells homogenously, no bubbles, holes, or inhomogeneities of any type were reported. From left to right panel a) reports Hep3Gel, GEL, and ALG, while panel b) reports ALG, GEL, and Hep3Gel. c) Shows hydrogels after being extracted from multi-well plates. Scalebars correspond to 1 cm

Supplementary Figure 11

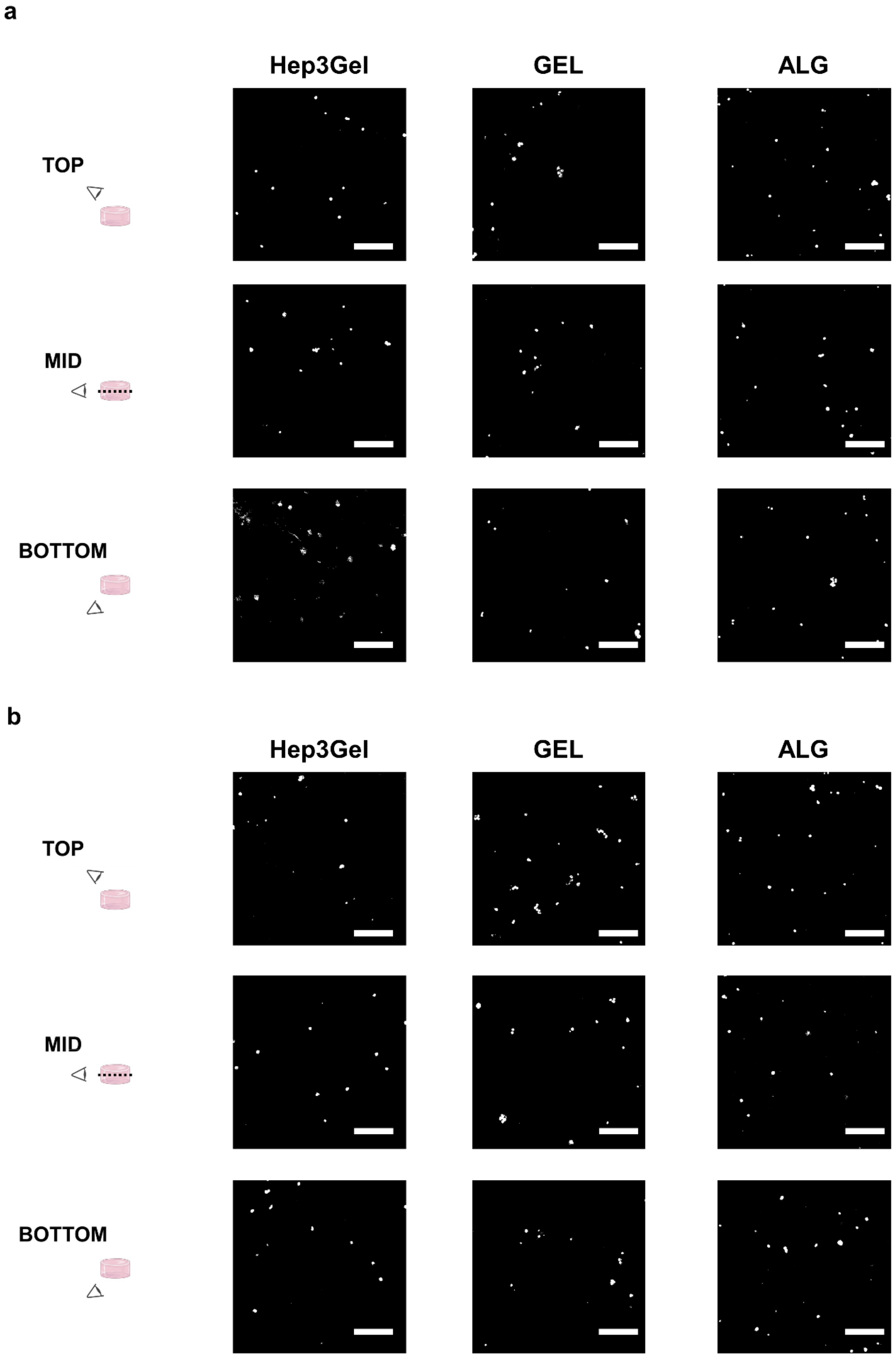

Figure S11 Homogeneous distribution of cells within the material. Distribution of cells in the top, middle, and bottom layer of Hep3Gel, GEL, and ALG before (a) and after (b) the gel-point. Original images reported cells colored in blue with Hoechst 3342, here are reported the 8-bit black and white images exploited to carry out the measurements with FIJI, aiming to improve the readability of the pictures. The scale bars correspond to 100 µm.
